# Nigericin and Geldanamycin Are Phytotoxic Specialized Metabolites Produced by the Plant Pathogen *Streptomyces* sp. 11-1-2

**DOI:** 10.1128/spectrum.02314-21

**Published:** 2022-02-28

**Authors:** Gustavo A. Díaz-Cruz, Jingyu Liu, Kapil Tahlan, Dawn R. D. Bignell

**Affiliations:** a Department of Biology, Memorial University of Newfoundland, St. John’s, Newfoundland and Labrador, Canada; USDA - San Joaquin Valley Agricultural Sciences Center

**Keywords:** phytotoxins, molecular networking, biosynthetic gene clusters, specialized metabolites, *Streptomyces*, plant pathogens

## Abstract

*Streptomyces* bacteria are a key source of microbial specialized metabolites with useful applications in medicine and agriculture. In addition, some species are important plant pathogens and cause diseases such as potato scab, which reduces the quality and market value of affected potato crops. Most scab-associated *Streptomyces* spp. produce the phytotoxic metabolite thaxtomin A as the principal pathogenicity factor. However, recent reports have described scab-causing strains that do not produce thaxtomin A, but instead produce other phytotoxins that are thought to contribute to plant host infection and symptom development. *Streptomyces* sp. 11-1-2 is a highly pathogenic strain that was originally isolated from a scab symptomatic potato tuber in Newfoundland, Canada. The strain secretes one or more phytotoxic compounds of unknown identity, and it is hypothesized that these compounds serve as virulence factors for this organism. We analyzed the genome sequence of *Streptomyces* sp. 11-1-2 and found biosynthetic gene clusters for producing the known herbicidal compounds nigericin and geldanamycin. Phytotoxic culture extracts were analyzed using liquid chromatography-coupled tandem mass spectrometry and molecular networking, and this confirmed the production of both compounds by *Streptomyces* sp. 11-1-2 along with other, potentially related metabolites. The biosynthesis of both metabolites was found to be suppressed by the addition of *N*-acetylglucosamine to the culture medium, and pure nigericin and geldanamycin were able to exhibit phytotoxic effects against both radish seedlings and potato tuber tissue. Furthermore, the coadministration of the two compounds produced greater phytotoxic effects against potato tuber tissue than administration of each compound alone.

**IMPORTANCE** Plant pathogens use a variety of mechanisms, including the production of phytotoxic specialized metabolites, to establish an infection of host tissue. Although thaxtomin A is considered the key phytotoxin involved in the development of potato scab disease, there is increasing evidence that other phytotoxins can play a role in disease development in some instances. In this study, we show that the highly pathogenic *Streptomyces* sp. 11-1-2 is capable of producing nigericin and geldanamycin, which individually and combined can cause significant damage to potato tuber tissue and radish seedlings. Our results suggest that the pathogenic phenotype of *Streptomyces* sp. 11-1-2 is due in part to the production of these specialized metabolites. As the biological activity of nigericin and geldanamycin is vastly different from the proposed activity of thaxtomin A against plants, the secretion of these compounds may represent a novel mechanism of plant pathogenicity exhibited by some *Streptomyces* species.

## INTRODUCTION

Members of the genus *Streptomyces* are widely regarded as one of the main natural sources of antibiotics and other medically relevant compounds, and they also have an important role in microbial communities associated with healthy soils and good plant development. The production of antimicrobial compounds has made several species of interest for agricultural purposes, as their presence results in suppression of plant pathogens and the diseases that they cause (1–4). However, some *Streptomyces* species are capable of causing plant diseases, of which scab disease of potato (Solanum tuberosum) is considered the most important. This disease is characterized by the development of superficial, raised, and/or pitted lesions that can cover a significant proportion of the tuber surface, and these lesions negatively affect the quality and market value of the potato crop (5, 6). Streptomyces scabiei (syn. *S. scabies*) was the first species to be described as a causal agent of potato scab, although multiple surveys across the world have resulted in the identification of several species and strains with phytopathogenic capabilities. Some of these other relevant species include Streptomyces turgidiscabies, Streptomyces acidiscabies, and Streptomyces europaeiscabiei, which are found throughout different potato-growing regions of the world (7).

Like other members of the *Streptomyces* genus, *S. scabiei* has the potential to produce a diverse array of specialized metabolites, but only a few have been determined to exhibit phytotoxic activity. Thaxtomin A, a 2,5-diketopiperazine, has been shown to be essential for scab disease development by S. scabiei, thus making it the principal pathogenicity determinant of this organism (8, 9). In addition to *S. scabiei*, other pathogenic species, such as S. turgidiscabies, S. acidiscabies, and S. europaeiscabiei, also produce thaxtomin A as the main pathogenicity factor (7). The biosynthesis of thaxtomin A in these species involves a gene cluster composed of seven open reading frames, of which six (*txtA*, *txtB*, *txtC*, *txtD*, *txtE*, *txtH*) play a direct role in metabolite biosynthesis, and one (*txtR*) functions in regulating metabolite production (10–14). This biosynthetic gene cluster (BGC) is highly conserved among scab-causing pathogens (15, 16) and is widely used for the detection and quantification of scab pathogens in soils using PCR (17–21). However, recent reports suggest that *Streptomyces* spp. that do not produce thaxtomin A are capable of causing scab disease symptoms on potato tubers, and this has been attributed to the production of other specialized metabolites with phytotoxic activity. Notably, *Streptomyces* sp. GK18 was isolated from a potato tuber exhibiting deep-pitted lesions, and this strain was subsequently shown to produce the 18-membered macrolide borrelidin. Pure borrelidin was shown to induce the same symptoms on potato tuber tissue as spores of the GK18 strain, suggesting that borrelidin is directly involved in scab disease development by this strain (22). Similarly, a Streptomyces niveiscabiei strain isolated from a pitted lesion was shown to produce the polyketide desmethylmensacarcin, and the pure compound was also more active at causing deep necrotic lesions on potato tubers than thaxtomin A (23, 24). Moreover, a survey conducted in Central Europe reported pathogenic isolates missing the *txtAB* genes from the thaxtomin BGC, suggesting that other unidentified factors are responsible for scab development (25).

A survey conducted in Newfoundland, Canada between 2011 and 2012 (26) reported the isolation of multiple pathogenic *Streptomyces* strains from scab-infected potatoes, one of which (*Streptomyces* sp. 11-1-2, herein referred to as 11-1-2) was found to produce one or more highly phytotoxic metabolites that were not thaxtomin A or borrelidin (26). The objective of the current study was to further investigate the phytotoxic compound(s) produced by 11-1-2 using genomic analyses, liquid chromatography-coupled mass spectrometry (LC-MS), and untargeted liquid chromatography-coupled tandem mass spectrometry (LC-MS/MS)-based metabolomics analysis. Using this approach, we identified nigericin and geldanamycin in 11-1-2 extracts, both of which are phytotoxic at low amounts and exhibit synergistic effects in promoting damage to potato tuber tissue. Further, we provide evidence that compounds related to these metabolites are also produced by *Streptomyces* sp. 11-1-2 and may contribute to the phytotoxic activity of this strain.

## RESULTS AND DISCUSSION

### *Streptomyces* sp. 11-1-2 is phylogenetically and metabolically distinct from other plant-pathogenic *Streptomyces* spp.

**FIG 1:**
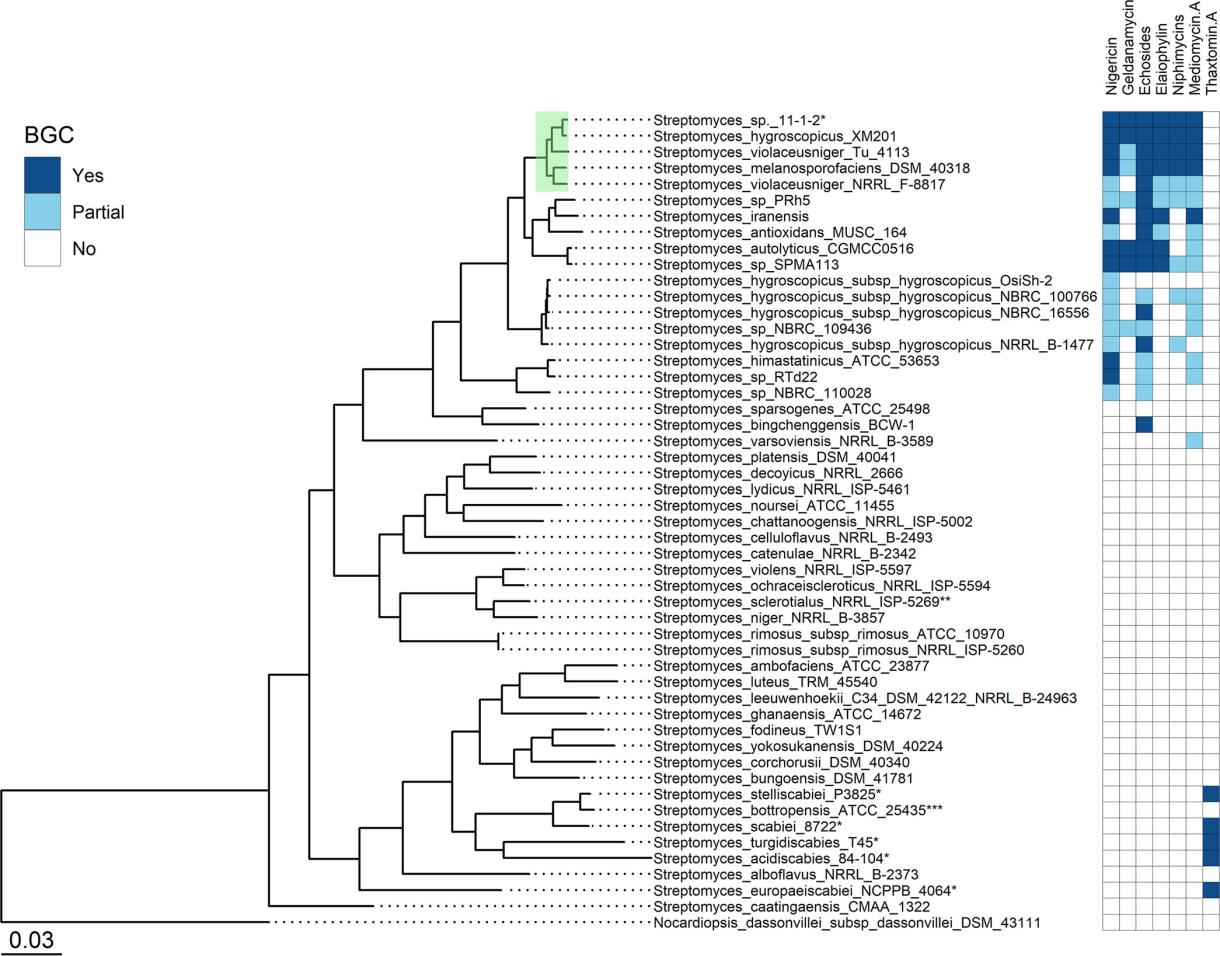
Maximum-likelihood multilocus species tree showing the phylogenetic relationship between *Streptomyces* sp. 11-1-2 and other *Streptomyces* species. *Streptomyces* sp. 11-1-2 localized to the clade that is highlighted in green and consists of strains with an average nucleotide identity of >95%. Plant-pathogenic strains are indicated with “*”. Strains for which the antiSMASH analysis did not show results are indicated with “**”. For Streptomyces bottropensis, only some strains of this species are plant pathogenic, “***”. The presence, absence, and partial presence of selected biosynthetic gene clusters are indicated in the heatmap. “Partial” correspond to BGCs not fully detected by antiSMASH due to genome completeness level or partial matches (<85% of similarity) to the BGC of the respective compound.

Initial work on the characterization of the 11-1-2 strain revealed that it is not closely related to the known scab pathogens *S. scabiei*, *S. acidiscabies*, *S. turgidiscabies*, or *S. europaeiscabiei*, suggesting that it belongs to a novel group of pathogenic species. To further elucidate the phylogenetic placement of 11-1-2, a multilocus species tree was constructed using the online tool autoMLST (27). The analysis revealed that 11-1-2 is highly similar to Streptomyces hygroscopicus strain XM201 (Fig. 1), sharing over 98% of the average nucleotide identity (Table S1). S. hygroscopicus XM201 was isolated from soil in China and has been reported as a source of different specialized metabolites (28–30). Other closely related strains include the soil-isolated Streptomyces violaceusniger Tü 4113 (31), Streptomyces melanosporofaciens DSM 40318, and *S. violaceusniger* NRRL F-8817. Type strains for these species have been grouped in the *S*. *violaceusniger* subclade, which in turn is part of the larger *S*. *hygroscopicus* clade (32, 33). Recently, an analysis of the *Streptomyces* pan-genome confirmed that 11-1-2 and *S. hygroscopicus* XM201 are closely related to each other and that both strains share more similarity at the genomic level with S. violaceusniger than with *S. hygroscopicus*, suggesting that they should be reclassified as *S. violaceusniger* (34). Plant-pathogenic *Streptomyces* strains are usually located within or near the *S. scabiei* clade. They often share some genomic traits such as thaxtomin A biosynthesis and the presence of other virulence genes, such as *nec1* and/or *tomA* (35–37). In contrast, 11-1-2 does not contain any of these markers (26), and it is not closely related to the classic scab-inducing strains (Fig. 1), which suggests that 11-1-2 is the first member of the *S. violaceusniger* subclade with plant-pathogenic capabilities.

To determine the potential metabolites responsible for the phytotoxic activity recorded previously, the genome sequence of 11-1-2 (38) was analyzed for the presence of specialized metabolite BGCs using antiSMASH 6.0 (39). The analysis determined the presence of 51 regions with various degrees of similarity to known BGCs (Table S2). Six of the regions are highly similar (≥85%) to BGCs that produce compounds with known antifungal, antimicrobial, and/or cytotoxic activities, i.e., nigericin, geldanamycin, echosides, elaiophylin, niphimycins, and mediomycin A (40–48). Interestingly, two of these, nigericin and geldanamycin, have been reported to exhibit herbicidal activity against some plants, including garden cress, cucumber, tomato, soybean, and wheat (41, 49). The other strains in the phylogenetic tree were also evaluated using antiSMASH to determine the presence of these six BGCs in their genomes (accession numbers available in Table S3). The comparison provided further evidence for the close relationship between 11-1-2 and *S. hygroscopicus* XM201, given that these strains both harbor all six of the BGCs (Fig. 1). Furthermore, the BGCs for production of nigericin, echosides, elaiophylin, niphimycins, and mediomycin A appear to constitute a “core” of BGCs that are conserved in the species that are most closely related to the 11-1-2 strain (Fig. 1). It has been reported that strains of *S. hygroscopicus* can produce different combinations of these compounds, with geldanamycin and nigericin commonly being coproduced (40–45). When the presence of BGCs across the *Streptomyces* genus is considered, geldanamycin and geldanamycin-like clusters are often found associated with nigericin clusters; however, neither of these clusters is found in genomes containing the thaxtomin BGC (Fig. S1).

**FIG 2:**
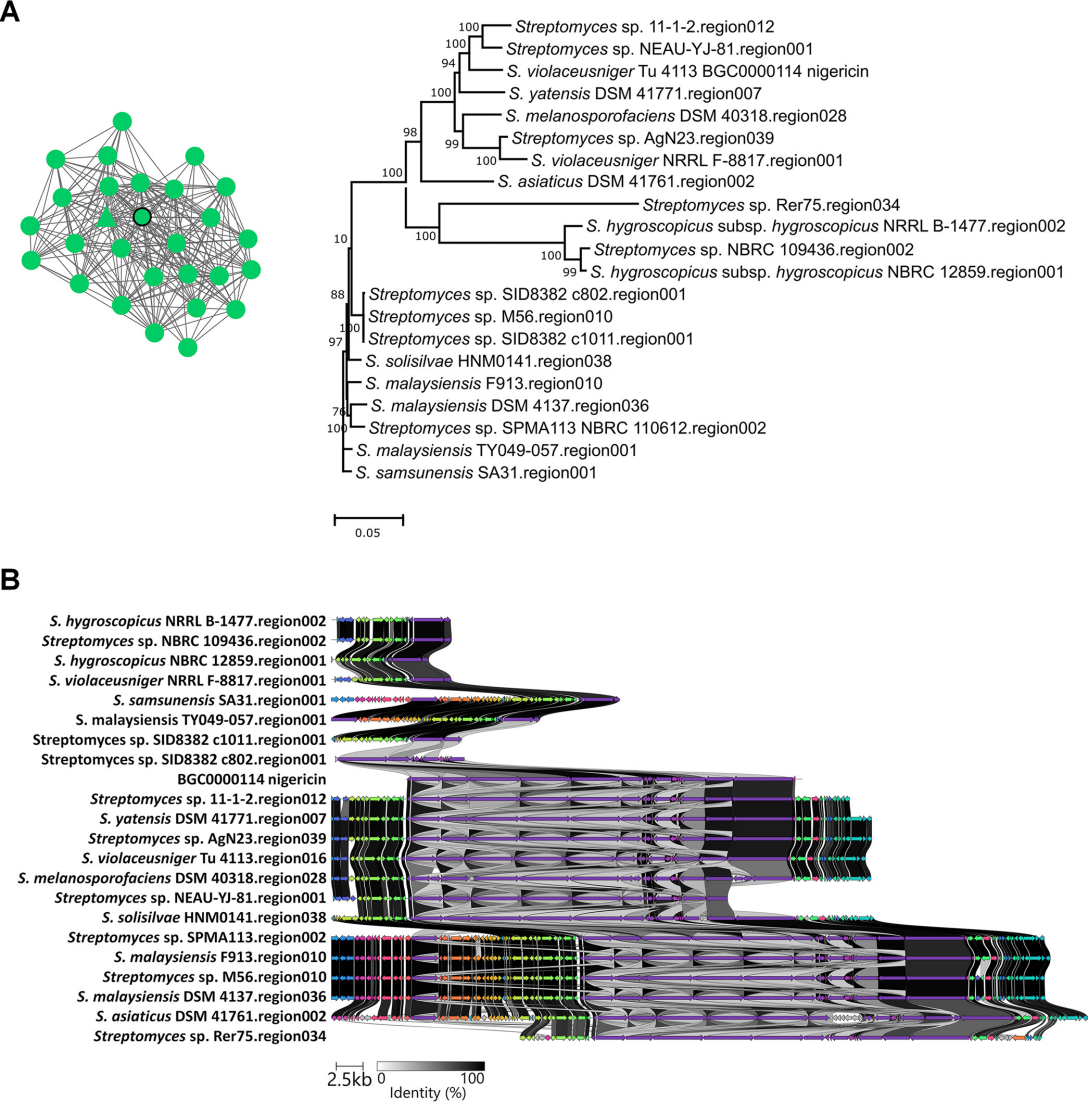
Large-scale analysis of nigericin BGCs from *Streptomyces* spp. (A) The similarity network of *Streptomyces* sp. 11-1-2 region 012 (nigericin BGC) and BGCs from other *Streptomyces* species (left), and their evolutionary relationships within the GCF (right). The triangular node within the network represents *Streptomyces* sp. 11-1-2 region 012, and the circular nodes represent known BGCs from the MIBiG database (outlined in thick black) and BGCs from other *Streptomyces* spp. Bootstrap values of ≥50% are shown at the respective branch points and are based on 1,000 repetitions. (B) Alignment of the *Streptomyces* sp. 11-1-2 region 012 with the known nigericin BGC (BGC0000114) from *S. violaceusniger* Tu 4113 and other BGCs within the same GCF. Genes colored the same belong to the same functional group, and homologues are linked by shaded areas that indicate the % identity.

**FIG 3:**
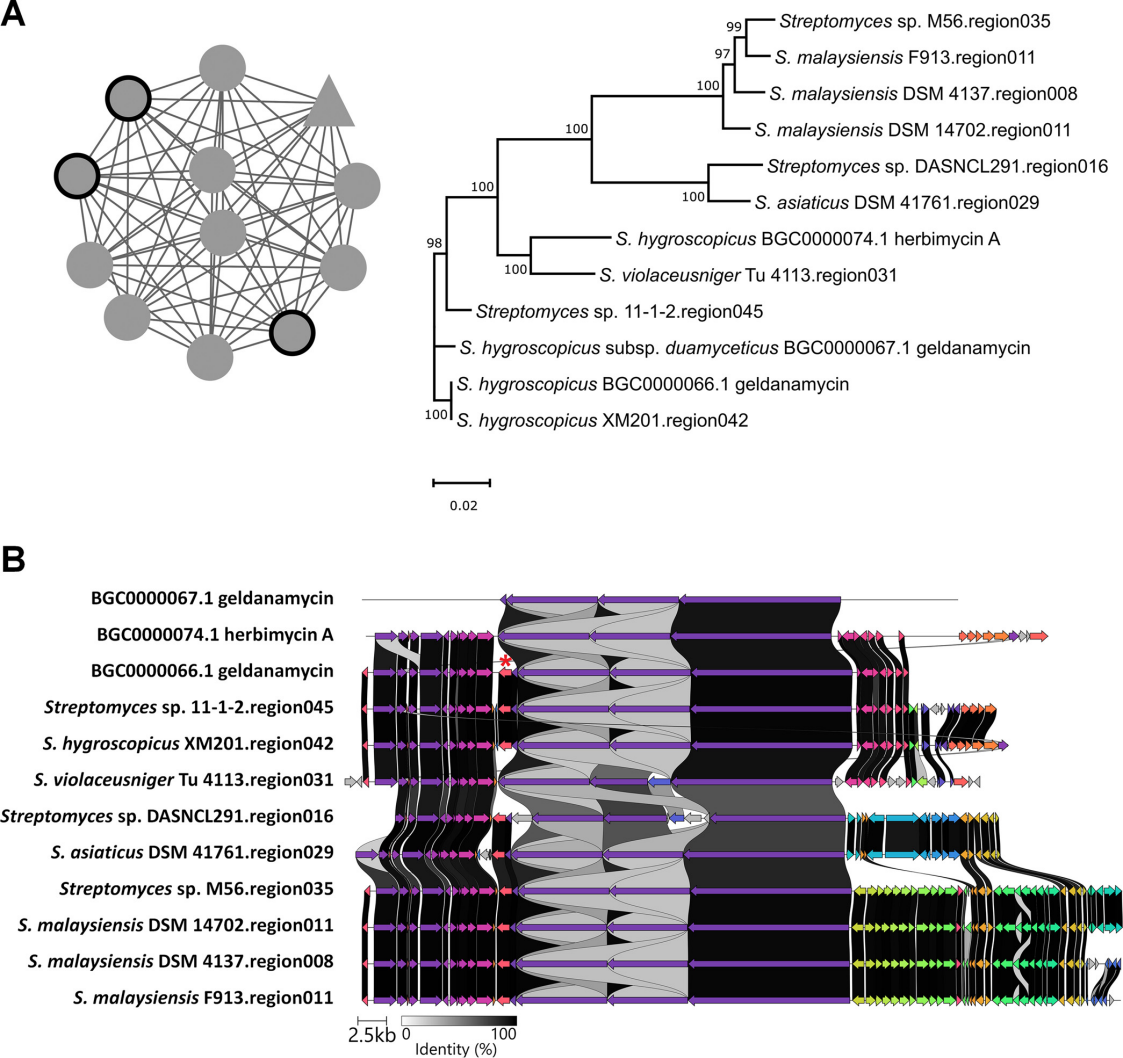
Large-scale analysis of geldanamycin BGCs from *Streptomyces* spp. (A) The similarity network of *Streptomyces* sp. 11-1-2 region 045 (geldanamycin BGC) and BGCs from other *Streptomyces* species (left), and their evolutionary relationships within the GCF (right). The triangular node within the network represents *Streptomyces* sp. 11-1-2 region 045, and the circular nodes represent known BGCs from the MIBiG database (outlined in thick black) and BGCs from other *Streptomyces* spp. Bootstrap values of ≥50% are shown at the respective branch points and are based on 1,000 repetitions. (B) Alignment of *Streptomyces* sp. 11-1-2 region 045 with the known geldanamycin BGC (BGC0000066.1), the known herbimycin BGC (BGC0000074.1) from *S. hygroscopicus*, and other BGCs within the same GCF. Genes colored the same belong to the same functional group, and homologues are linked by shaded areas that indicate the percent identity. The *gdmM* gene from the geldanamycin BGC that is conserved in the 11-1-2 region 045 and is missing from the herbimycin BGC is indicated with a red asterisk above the gene.

To further explore the conservation and evolution of the nigericin and geldanamycin BGCs among *Streptomyces* spp., the BiG-SCAPE computational tool was employed (50). BiG-SCAPE (biosynthetic gene similarity clustering and prospecting engine) uses antiSMASH-detected BGCs to generate sequence similarity networks and group them into gene cluster families (GCFs) along with reference BGCs from the MIBiG (minimum information about a biosynthetic gene cluster) database, and it elucidates the evolutionary relationships of the BGCs within each GCF. Using BGCs obtained from 2,136 *Streptomyces* genomes, BiG-SCAPE generated a similarity network for the *Streptomyces* sp. 11-1-2 region 012 (the predicted nigericin BGC; Table S2) that consisted of 28 BGCs (Fig. 2A), and these 28 BGCs were grouped into two distinct GCFs. Phylogenetic analysis of the GCF containing the predicted 11-1-2 nigericin BGC (Fig. 2A) showed that this gene cluster is most closely related to the *Streptomyces* sp. NEAU-YJ-81 region 001 and the known nigericin BGC (BGC0000114) from *S. violaceusniger* Tu 4113. An alignment of the BGCs from the GCF tree revealed that the gene content and architecture of the nigericin BGC are highly conserved among different *Streptomyces* spp., including the 11-1-2 strain (Fig. 2B). This supports the notion that the 11-1-2 strain is capable of biosynthesizing nigericin. It should be pointed out that the nigericin BGC comparison was affected by the assembly level of the genome sequences available in the public database. Although the BGC appears to be incomplete in some strains (Fig. 2B), this is most likely due to the presence of gaps in the genome sequences of these organisms. The similarity network for the 11-1-2 region 045 (the predicted geldanamycin BGC; Table S2) consisted of 12 BGCs that were grouped into a single GCF, and phylogenetic analysis revealed that the 11-1-2 BGC does not cluster with the other BGCs in the GCF (Fig. 3A). A comparison of the gene content and organization (Fig. 3B) revealed that the 11-1-2 BGC is most similar to the geldanamycin BGC from *S. hygroscopicus* XM201 (region 042). Notably, the 11-1-2 BGC contains a gene (*CGL_43245*) that is homologous to *gdmM* from the *S. hygroscopicus* geldanamycin BGC (BGC0000066.1). This gene is absent from the closely related herbimycin BGC (BGC0000074.1) (Fig. 3B) and is proposed to function in an oxidation step that occurs during geldanamycin biosynthesis but not during herbimycin biosynthesis (51). Thus, the presence of this gene in the 11-1-2 BGC suggests that 11-1-2 produces geldanamycin rather than herbimycin.

### The production of phytotoxic compounds by *Streptomyces* sp. 11-1-2 is dependent on medium composition.

**FIG 4:**
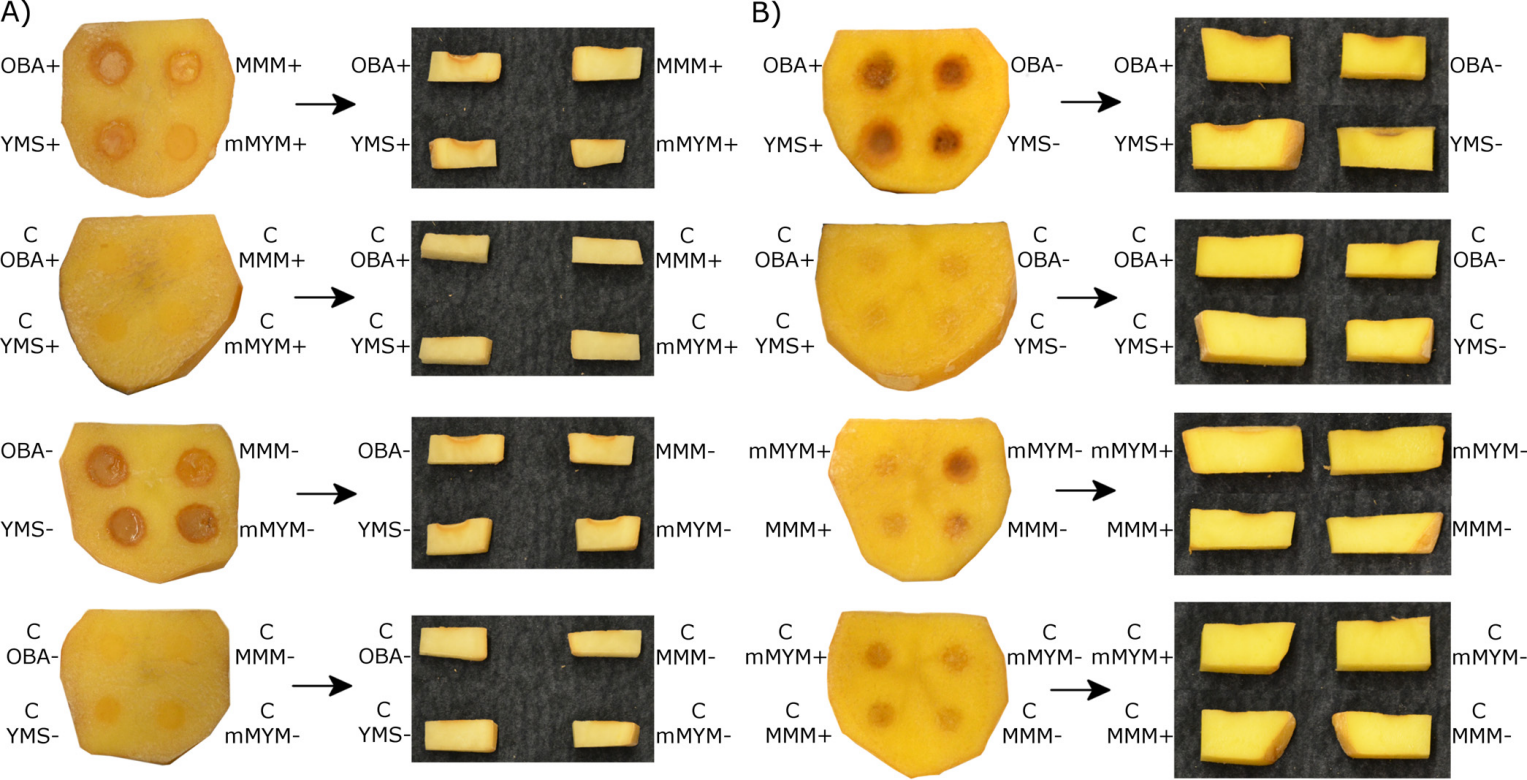
Phytotoxic activity of agar cores (A) and organic culture extracts (B) on excised potato tuber tissue. Each tuber slice contained four agar cores or four disks wetted with 20 μL of culture extract. The 11-1-2 strain was cultured on OBA, YMS, MMM, and mMYM media, with (+) and without (−) 50 mM NAG for 14 days. The agar cores were obtained directly from the agar plates, while the extracts were prepared from one whole plate of each medium. Names of culture media preceded by a “C” are cores or extracts from control (noninoculated) media. Photos were taken at 7 days after inoculation and show the top and side views of the tuber slices at the inoculation sites. Each slice had three replicates per experiment. The assay was performed twice with three biological replicates per treatment in each assay, with similar results obtained each time.

To identify the conditions that promote the production of phytotoxic compounds by the 11-1-2 strain, we cultured the strain on four different agar media containing or lacking *N*-acetylglucosamine (NAG). The addition of NAG to culture media has differential effects on *Streptomyces* morphological development and specialized metabolite production, depending on the type of medium used. Specifically, addition to nutrient-rich culture media typically results in disruption of sporulation in favor of a vegetative state, and the production of specialized metabolites can be either stimulated or suppressed (52, 53). In contrast, addition of NAG to nutrient-poor media generally enables sporulation and stimulates specialized metabolite production (54). Two of the media used, modified maltose-yeast extract-malt extract agar (mMYM [55]) and yeast extract-malt extract-starch agar (YMS [56]), are nutrient-rich media and have been used for the production of specialized metabolites by other *Streptomyces* spp. Oat bran agar (OBA [57]) is a plant-based medium previously shown to support phytotoxin production by the 11-1-2 strain (26), and minimal medium with mannitol (MMM [58]) is a simple, defined medium that supports growth and sporulation by *Streptomyces* spp. and has been used in other studies to characterize the effects of NAG on morphology and specialized metabolite production (52, 54). Following incubation of the 11-1-2 strain on the different media, agar cores were removed from the plates and were placed onto potato tuber tissue slices, after which the slices were incubated in a moist chamber for 7 days. As shown in Fig. 4A, the cores from the 11-1-2-inoculated plates caused pitting, softening, and browning of the tuber tissue around the contact area, while cores from the uninoculated control plates had no effect. The OBA and YMS cores from the inoculated plates caused similar tissue damage regardless of whether or not NAG was present in the medium. In contrast, cores from the mMYM and MMM plates lacking NAG caused greater damage than those from plates containing NAG, suggesting that NAG influences the production of the phytotoxic compounds in these media.

Next, the agar plates were extracted with ethyl acetate, and the resulting culture extracts were evaluated for phytotoxicity using the potato tuber slice bioassay. As shown in Fig. 4B, the extracts prepared from the 11-1-2-inoculated OBA and YMS with or without NAG plates all caused pitting and browning of the tuber tissue in a manner similar to that of the corresponding agar cores (Fig. 4A), though the browning was darker with the extracts. The extract from the inoculated mMYM without NAG medium showed some pitting and browning, though the effect was less severe than that with the OBA and YMS extracts. In contrast, the extracts prepared from the mMYM with NAG and MMM with or without NAG plates did not cause any damage that differed from that of the control extracts prepared from the uninoculated media (Fig. 4B).

**FIG 5:**
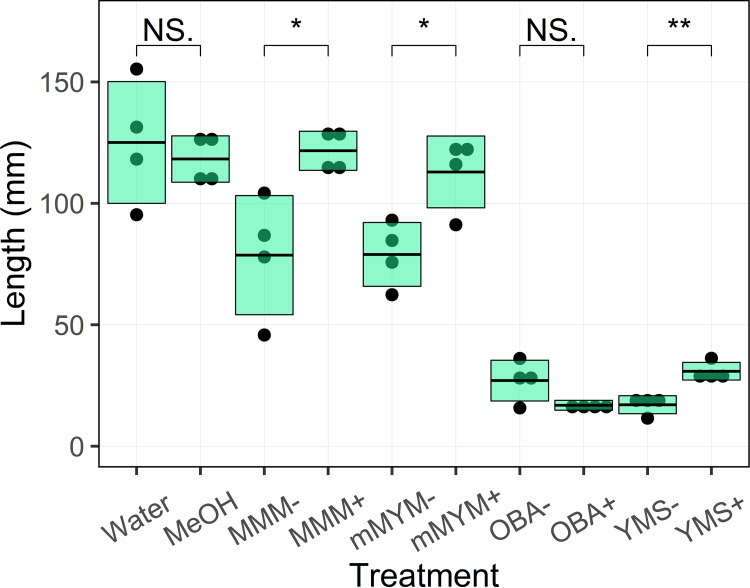
Phytotoxic activity of organic culture extracts on radish seedlings. Extracts were prepared from 14-day-old plate cultures of 11-1-2 grown on OBA, YMS, MMM, and mMYM media, with (+) and without (−) 50 mM NAG. Each box represents the average length of four seedlings plus or minus one standard deviation. To evaluate how the addition of NAG to the culture medium affected the phytotoxic activity against the seedlings, the treatments were analyzed using the Student’s *t* test in pairs (NS., not significant; *, *P ≤ *0.05; **, *P ≤ *0.01). The assay was performed three times, with similar results obtained each time.

We also evaluated the organic culture extracts using a radish seedling bioassay, since such an assay was previously used to detect the phytotoxic activity of the 11-1-2 strain (26). The results of the assay were in agreement with those of the potato tuber tissue assay in that the OBA and YMS extracts showed greater phytotoxic activity than the mMYM and MMM extracts (Fig. 5). The OBA and YMS extracts all caused severe stunting of the radish seedlings compared to the water and solvent controls, and interestingly, the presence of NAG in the YMS medium caused a significant reduction in activity compared to that of the same medium without NAG. NAG also had a suppressive effect on the phytotoxic activity when the 11-1-2 strain was cultured on mMYM and MMM, with the NAG+ extracts showing little effect on the seedlings compared to the water and solvent controls (Fig. 5).

Overall, the results of the two bioassays demonstrate that YMS and OBA both promote the production of high levels of the phytotoxic compound(s) by the 11-1-2 strain compared to MMM and mMYM and that NAG has a negative regulatory effect on the phytotoxic activity in some media, as well as on morphological development (Fig. S2). It has been reported that NAG acts as a signal for the activation of the global regulator DasR, which in turn can either activate (under nutrient-poor conditions) or repress (under nutrient-rich conditions) the expression of genes associated with morphological development and specialized metabolite production (52). The DNA binding site of DasR has been characterized experimentally, and *in silico* analysis of the 11-1-2 genome sequence identified several possible binding sites (Table S4). Among the predicted sites are those upstream of chitinase and NAG metabolism-associated genes, which are known targets of DasR in other *Streptomyces* spp. (54, 59). Some of the predicted binding sites are associated with genes encoding GntR, TetR/AraC, LysR, and LuxR transcriptional regulators, whereas none are located within the BGCs identified in the antiSMASH results. Therefore, it is possible that DasR indirectly regulates the production of the phytotoxic compounds through one or more global regulatory proteins, and future studies will aim to further investigate this.

### *Streptomyces* sp. 11-1-2 produces the herbicidal compounds geldanamycin and nigericin.

To identify the phytotoxic compounds produced by 11-1-2, we subjected organic extracts prepared from YMS and mMYM (with or without NAG) plate cultures to untargeted LC-MS/MS analysis. The organic extracts from these media were selected based on the general differences in phytotoxic activity recorded in our previous bioassays. The resulting MS/MS spectral data were analyzed using the ion identity molecular networking (IIMN) module of the feature-based molecular networking (FBMN) workflow within the global natural products social molecular networking (GNPS) web platform (60–62). With this analysis, molecular networks of fragmentation spectra for the detected ion adducts can be generated, with nodes (each representing a single fragmentation spectrum) linked based on similarity in retention time, peak shape, and fragmentation patterns. The MS/MS spectra obtained were annotated by performing a GNPS spectral library search, which compares the experimental spectral data to a library of MS/MS reference spectra for known metabolites (62). Spectra with no matches to the library were analyzed using SIRIUS (63, 64), which provides a prediction of the molecular formula, and MetFrag, which compares the fragmentation pattern and molecular formula to annotate and classify molecule candidates by comparing them to the PubChem database (65).

**FIG 6:**
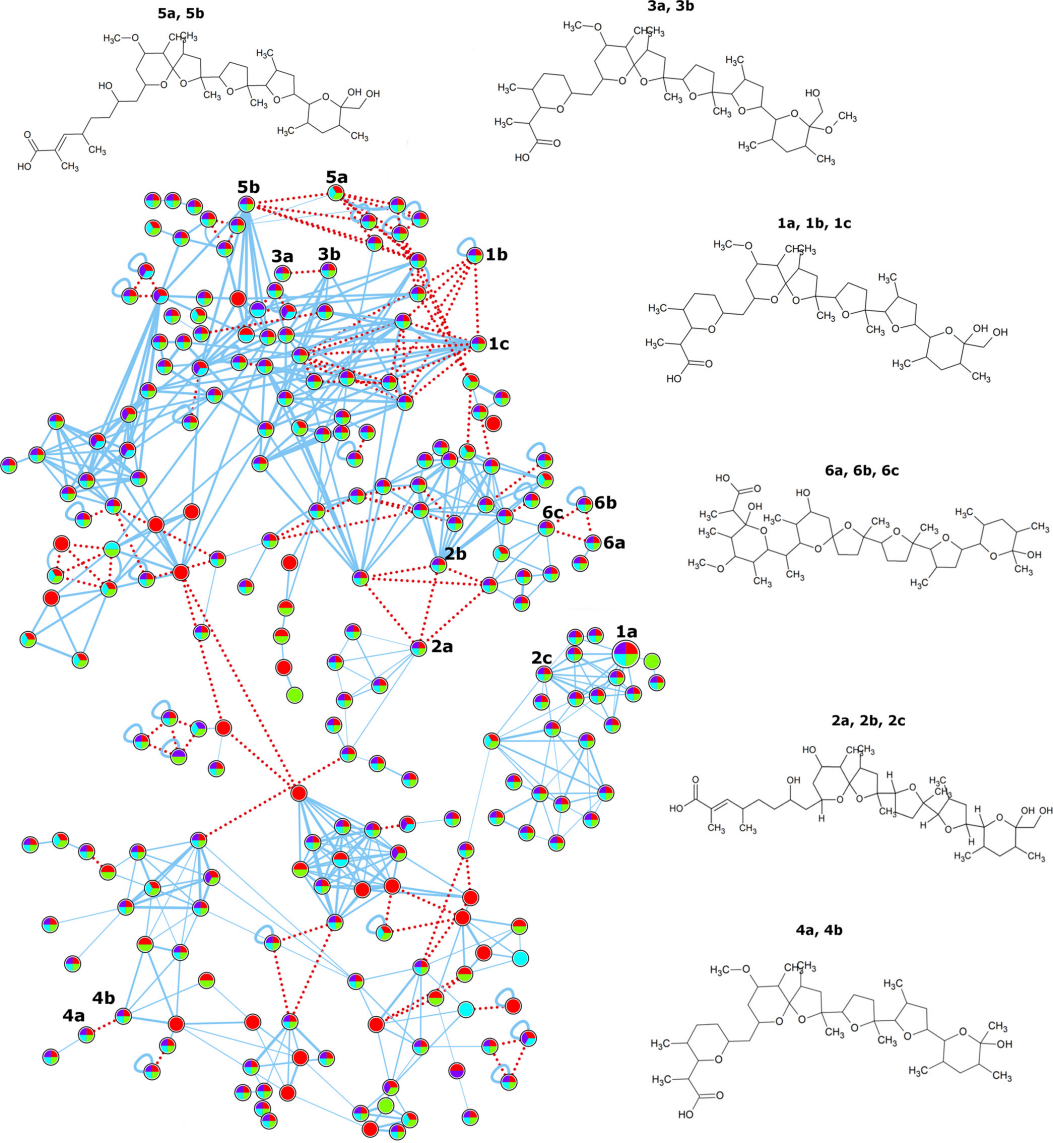
Molecular network for nigericin. Organic culture extracts were prepared from plate cultures of 11-1-2 grown on YMS and mMYM (with or without 50 mM NAG) for 14 days. The extracts were evaluated using LC-MS/MS, and the resulting spectral data were analyzed using ion identity molecular networking. Each node in the network represents one fragmentation spectrum, and the structures of the predicted compounds listed in Table 1 are shown and numbered to match the corresponding nodes. Nodes are linked by a blue line if the cosine score is >0.7 and there are at least six matched fragment ions, thus sharing MS/MS identity. The width of the line represents the score between two nodes (0.7 to 1.0). Nodes linked by a red dotted line share MS identity. Each node shows a pie chart that represents the presence of the compound in extracts obtained from the different culture media. Pie chart legend: purple, mMYM with NAG; red, mMYM without NAG; green, YMS with NAG; teal, YMS without NAG. Larger nodes represent matches to the GNPS spectral libraries.

**FIG 7:**
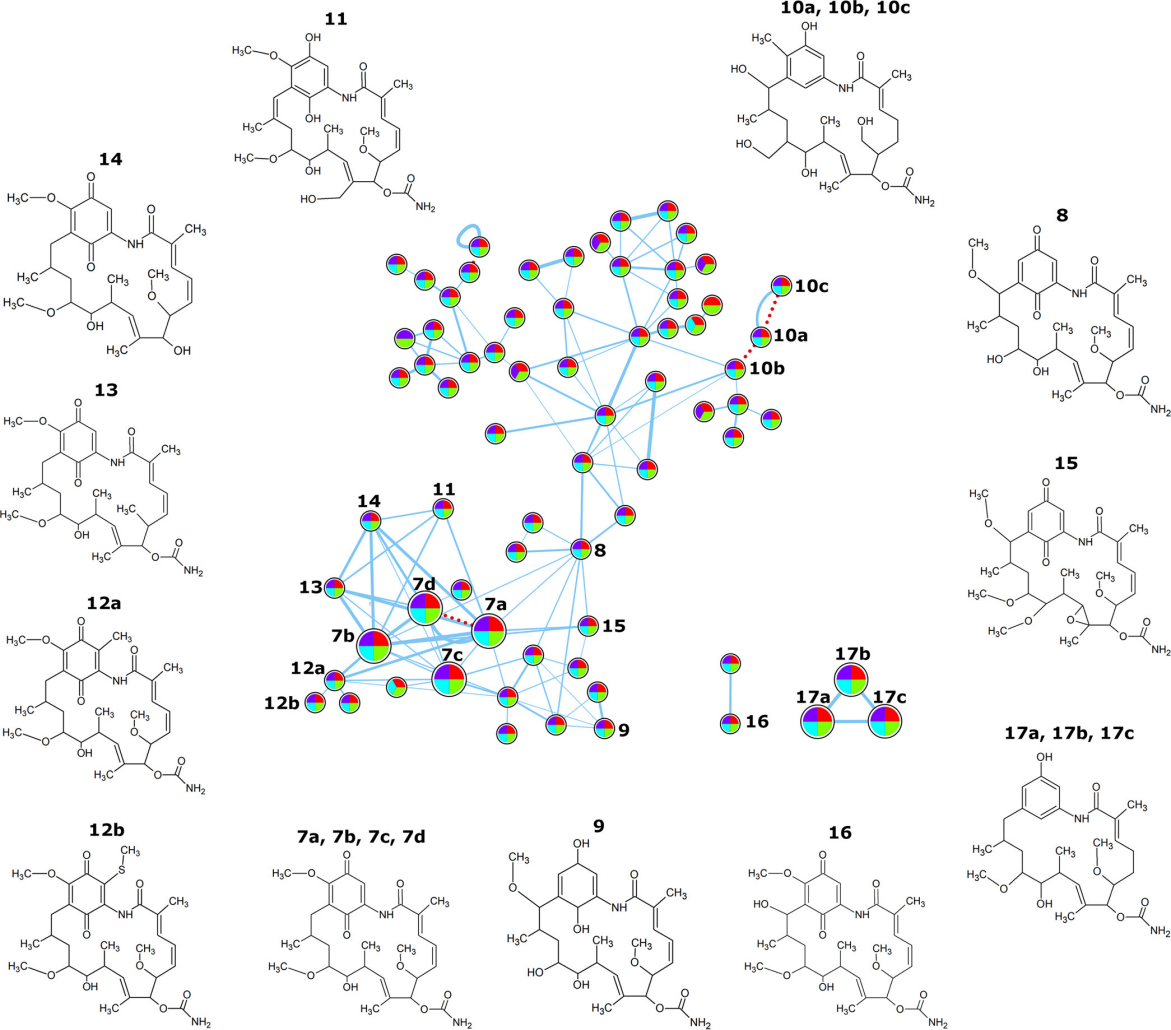
Molecular network for geldanamycin. Organic culture extracts were prepared from plates cultures of 11-1-2 grown on YMS and mMYM (with or without 50 mM NAG) for 14 days. The extracts were evaluated using LC-MS/MS, and the resulting spectral data were analyzed using ion identity molecular networking. The features of the network are as described in the Fig. 6 figure legend. The structures of the predicted compounds are shown.

Given that nigericin and geldanamycin are predicted to be produced by the 11-1-2 strain, and that these compounds have known herbicidal activity, we employed IIMN to analyze the MS/MS data for the presence of these metabolites in the extracts. Using this approach, we were able to annotate one molecular network containing a node with a spectral match to nigericin in the GNPS libraries (Fig. 6; Table 1). For nigericin, three different ion adducts were detected. In addition, we annotated one network with nodes having spectral matches to geldanamycin in the GNPS libraries (Fig. 7; Table 1).

**TABLE 1:** Summary of compounds associated with nigericin and geldanamycin obtained from the IIMN-FBMN analysis

Molecular formula^*a*^	Compound name^*b*^	Calculated mol wt (g/mol)	Adduct	*m/z*	Retention time (min)	Figure label^*c*^
C_40_H_68_O_11_	Nigericin*	725.0	[M-H]^−^	723.4683	13.6815	1a
			[M+Na]^+^	747.4641	13.6341	1b
			[M+NH_4_]^+^	742.5091	13.6315	1c
C_39_H_66_O_11_	*O*-demethylabierixin	710.9	[M+Na]^+^	733.4486	13.2231	2a
			[M+NH_4_]^+^	728.4932	13.2162	2b
			[M-H]^−^	709.4504	13.1907	2c
C_41_H_70_O_11_	29-*O*-methylabierixin	739.0	[M+Na]^+^	761.4821	13.9350	3a
			[M+NH_4_]^+^	756.5280	13.9400	3b
C_40_H_68_O_10_	Grisorixin	709.0	[M+Na]^+^	731.4717	12.8333	4a
			[M+NH_4_]^+^	726.5145	12.8081	4b
C_40_H_68_O_11_	Abierixin	725.0	[M+Na]^+^	747.4647	15.4556	5a
			[M+NH_4_]^+^	742.5098	15.5002	5b
C_41_H_70_O_12_	Mutalomycin	755.0	[M-H]^−^	753.4796	13.7183	6a
			[M+Na]^+^	777.4760	12.7979	6b
			[M-H_2_O+H]^+^	737.4870	12.7643	6c
C_29_H_40_N_2_O_9_	Geldanamycin*	560.6	[M-H]^−^	559.2657	11.2173	7a
			[M-H]^−^	559.2654	11.1868	7b
			[M-H]^−^	559.2656	9.6200	7c
			[M+Cl]^−^	595.2423	11.2024	7d
C_28_H_38_N_2_O_9_	Antibiotic TAN-420B	546.6	[M-H]^−^	545.2506	10.8291	8
C_28_H_42_N_2_O_9_	[(4E,6Z,8S,9S,10E,12S,13R,14S,16S,17R)-13,14,20,22-Tetrahydroxy-8,17-dimethoxy-4,10,12,16-tetramethyl-3-oxo-2-azabicyclo[16.3.1]docosa-1(21),4,6,10,18-pentaen-9-yl] carbamate	550.6	[M-H]^−^	549.2806	9.3509	9
C_29_H_44_N_2_O_8_	[(8R,9R,12S,13R,14R,16S,17R)-13,17,20-Trihydroxy-8,14-bis(hydroxymethyl)-4,10,12,16,19-pentamethyl-3-oxo-2-azabicyclo[16.3.1]docosa-1(22),4,10,18,20-pentaen-9-yl] carbamate	548.7	[M-H]^−^	547.3027	9.4444	10a
			[M-H_2_O+Cl]^−^	565.2744	9.2727	10b
			[M+FA]^−^	593.3081	9.4809	10c
C_29_H_40_N_2_O_10_	[(8S,9S,12S,13R,14S)-13,20,22-Trihydroxy-10-(hydroxymethyl)-8,14,19-trimethoxy-4,12,16-trimethyl-3-oxo-2-azabicyclo[16.3.1]docosa-1(21),4,6,10,16,18(22),19-heptaen-9-yl] carbamate	576.6	[M-H]^−^	575.2594	10.9951	11
C_30_H_42_N_2_O_9_	19-Methylgeldanamycin	574.7	[M-H]^−^	573.2798	9.5806	12a
C_30_H_42_N_2_O_9_S	19-S-Methylgeldanamycin	606.7	[M-H]^−^	605.2524	9.9423	12b
C_29_H_40_N_2_O_8_	6-Demethoxy-6-methylgeldanamycin	544.6	[M-H]^−^	543.2723	11.8121	13
C_28_H_39_NO_8_	7-*O*-descarbamoyl-7-hydroxygeldanamycin	517.6	[M-H]^−^	516.2596	11.1875	14
C_30_H_42_N_2_O_10_	8,9-Epoxyherbimycin A	590.7	[M-H]^−^	589.2768	11.1210	15
C_29_H_40_N_2_O_10_	15-Hydroxygeldanamycin	576.6	[M-H]^−^	575.2605	10.3256	16
C_28_H_42_N_2_O_7_	EH21A2/Autolytimycin*	518.6	[M-H]^−^	517.2915	9.3496	17

a The molecular formulas were obtained from analysis of the spectra using SIRIUS and MetFrag.

b Names with “*” were a match to the GNPS database. Names without “*” are the PubChem entry for the predictions estimated by MetFrag.

c The numbers in this column refer to the nodes labeled in Fig. 6 and 7.

Along with nigericin and geldanamycin, the accumulation of biosynthetic intermediates or derivatives of these compounds has been reported in other studies, and the presence of some of these in our extracts was also predicted based on the MS/MS data obtained (Table 1; Fig. 6 and 7). For example, abierixin and grisorixin are compounds that are closely related to nigericin and are coproduced with nigericin (66–68). Studies in other *Streptomyces* spp. suggest that these molecules along with *O*-demethylabierixin, which was also predicted to be present in our samples (Table 1; Fig. 6), may be intermediates in the biosynthesis of nigericin (69). Other nigericin-related compounds predicted from our data include 29-*O*-methylabierixin, which was first detected in *S. hygroscopicus* XM201 (28), and mutalomycin, initially detected in Streptomyces mutabilis NRRL 8088 (Table 1; Fig. 6) (70). Among the detected molecules that are predicted to be structurally related to geldanamycin is the antibiotic TAN-420B, which was originally obtained from strains of *S. hygroscopicus* and was reported to have weak antimicrobial activity (Table 1; Fig. 7) (71). Other geldanamycin-related compounds annotated in our samples (Table 1; Fig. 7) have previously been obtained by fermentation of *Streptomyces* strains (72–74). Two smaller, separate networks also contained geldanamycin-related compounds. The first one is predicted to match 15-hydroxygeldanamycin (75), while the other network contains EH21A2 (or autolytimycin) (76).

**FIG 8:**
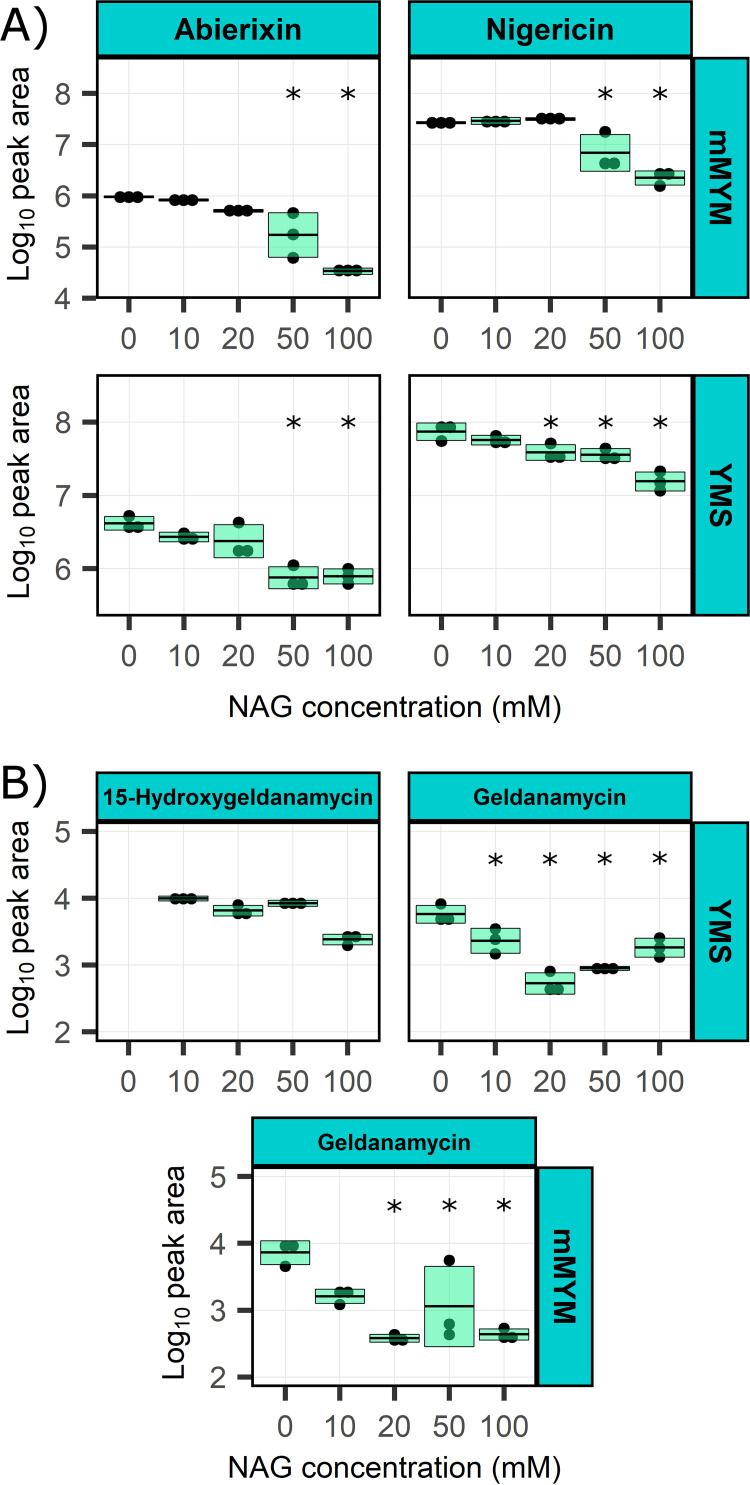
Effects of NAG on the production of nigericin and the related molecule abierixin (A) and of geldanamycin and the related molecule 15-hydroxygeldanamycin (B). Organic culture extracts were prepared from plate cultures of 11-1-2 grown for 14 days on YMS and mMYM supplemented with different concentrations of NAG. The extracts were evaluated using LC-MS (nigericin and abierixin) and RP-HPLC (geldanamycin and 15-hydroxygeldanamycin). The areas of the nigericin/abierixin and geldanamycin/15-hydroxygeldanamycin peaks were obtained using MestReNova and Chemstation, respectively, and were transformed using the log_10_ function. An ANOVA paired with Dunnett’s test was performed in Minitab, and media without NAG were used as control treatments for statistical analysis. Data boxes showing an asterisk (*) are statistically different from their respective control (*P* < 0.05). Each box represents the average peak area of three extracts plus or minus one standard deviation. The RP-HPLC analysis did not show a peak for 15-hydroxygeldanamcyin in extracts from YMS (0 mM NAG); thus, no statistical analysis was performed. A peak for 15-hydroxygeldanamycin was not observed for any of the mMYM with/without NAG extracts, and thus this graph was not included.

Since the phytotoxic activity of the 11-1-2 strain was found to be suppressed by NAG in some media, we wanted to determine whether NAG suppresses production of nigericin and geldanamycin by this strain. Thus, we tested the effects of different concentrations of NAG on the production of these metabolites in mMYM and YMS. In the case of nigericin, NAG concentrations of 50 and 100 mM in mMYM significantly reduced the metabolite production level, while production in YMS was significantly decreased starting at 20 mM NAG (Fig. 8A). Interestingly, a much smaller peak with the same *m/z* (742.5 [M+NH_4_]^+^) as nigericin but with a different retention time was also detected in the culture extracts (Fig. S3). A similar peak was previously observed in the cultures extracts of the nigericin producer *Streptomyces* sp. DSM4137 and is presumed to be abierixin (68), which was also predicted to be present based on our IIMN analysis (Fig. 6; Table 1). The peak area for this compound was much smaller than that of nigericin in both the mMYM and YMS culture extracts, but it showed a similar trend in terms of the suppressive effects of NAG (Fig. 8A). For geldanamycin, the addition of NAG significantly reduced the metabolite production levels starting at 20 mM NAG in both mMYM and YMS (Fig. 8B). Moreover, the addition of NAG to YMS yielded another prominent peak with a retention time different from that of geldanamycin (Fig. S4). LC-MS analysis of this peak revealed an *m/z* of 575.2 ([M-H]^−^), which together with our IIMN results (Fig. 7; Table 1) suggests the presence of 15-hydroxygeldanamycin, a geldanamycin derivative previously obtained by the bioconversion of geldanamycin (75). Interestingly, our analysis revealed that this compound is present only when NAG is added to YMS, and the amount produced remained relatively constant at NAG concentrations of 10 to 50 mM and decreased only at higher NAG concentrations (Fig. 8B). Quantification of the nigericin and geldanamycin production levels demonstrated that the 11-1-2 strain produces significantly higher amounts of nigericin than geldanamycin in both mMYM and YMS (Table 2). Furthermore, in the absence of NAG, the level of nigericin in the YMS culture extract was almost three times that in the mMYM extract, and this appeared to correlate with the relative phytotoxicity of these extracts in our bioassays (Fig. 4 and 5), suggesting that nigericin is a major contributor to the observed phytotoxicity of the 11-1-2 culture extracts.

**TABLE 2:** Quantification of nigericin and geldanamycin obtained from organic culture extracts

Medium	NAG (mM)	Nigericin (mM)^*a*^	Geldanamycin (mM)*^a^*
mMYM	0	11.90 ± 0.58	0.25 ± 0.09
	10	13.00 ± 1.92	0.05 ± 0.01
	20	14.02 ± 0.88	0.01 ± 0.00
	50	3.88 ± 3.42	0.07 ± 0.09
	100	1.02 ± 0.31	0.01 ± 0.00
YMS	0	33.44 ± 8.81	0.19 ± 0.06
	10	25.28 ± 3.71	0.08 ± 0.03
	20	17.40 ± 4.46	0.02 ± 0.01
	50	15.98 ± 3.25	0.03 ± 0.00
	100	7.07 ± 2.14	0.06 ± 0.02

a The values correspond to the average of three replicates plus or minus one standard deviation.

### Geldanamycin and nigericin exhibit phytotoxic activity against potato tissue and radish seedlings.

**FIG 9:**
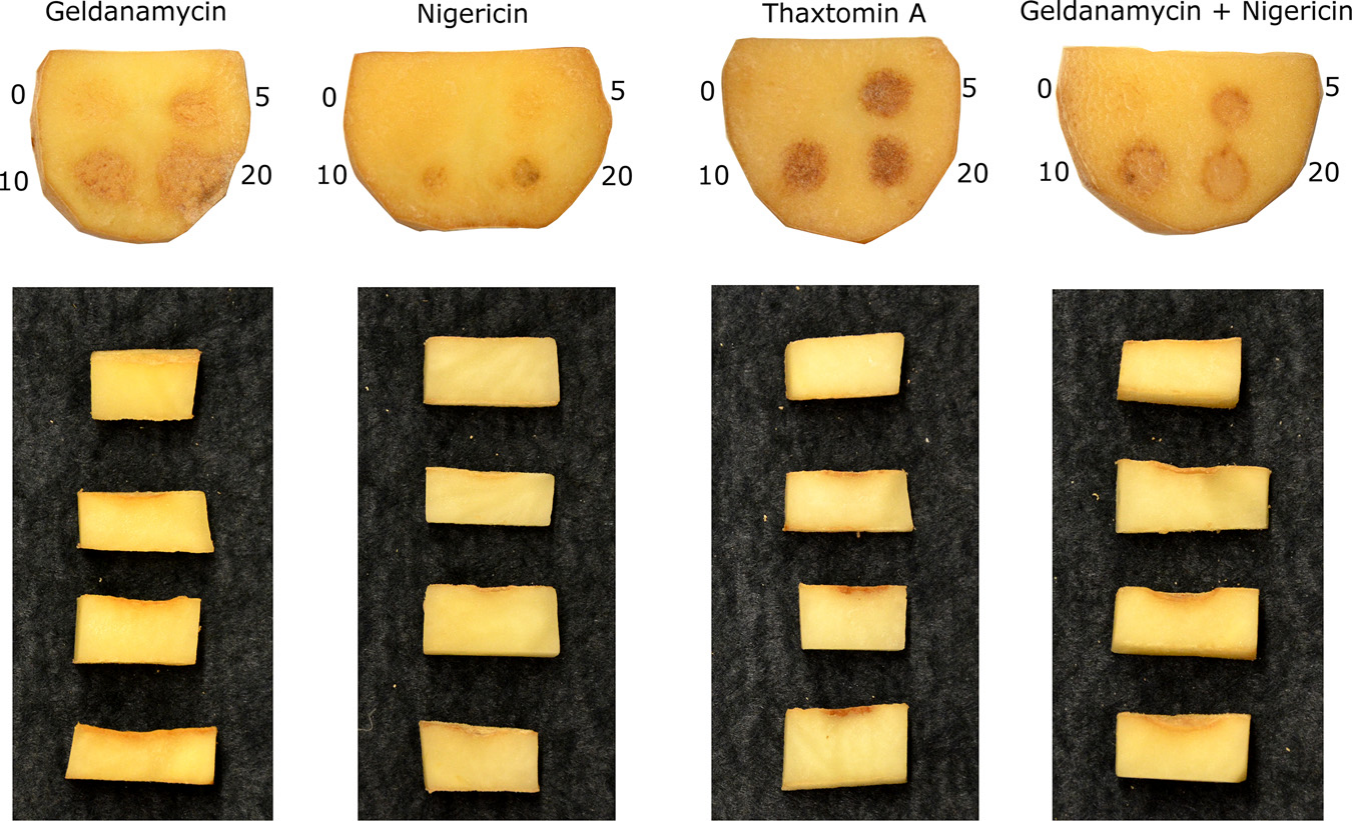
Phytotoxic effects of pure geldanamycin, nigericin, and thaxtomin A on potato tuber tissue. Each tuber slice contained four disks inoculated with 0 (control), 5, 10, and 20 nmol of the respective compound in a fixed volume of 20 μL. For the combination of geldanamycin with nigericin, each compound provided half of the amount reported, i.e., 5, 10, and 20 nmol had 2.5, 5, and 10 nmol of each compound. The assay was performed twice with three biological replicates per treatment in each assay, with similar results obtained each time.

**FIG 10:**
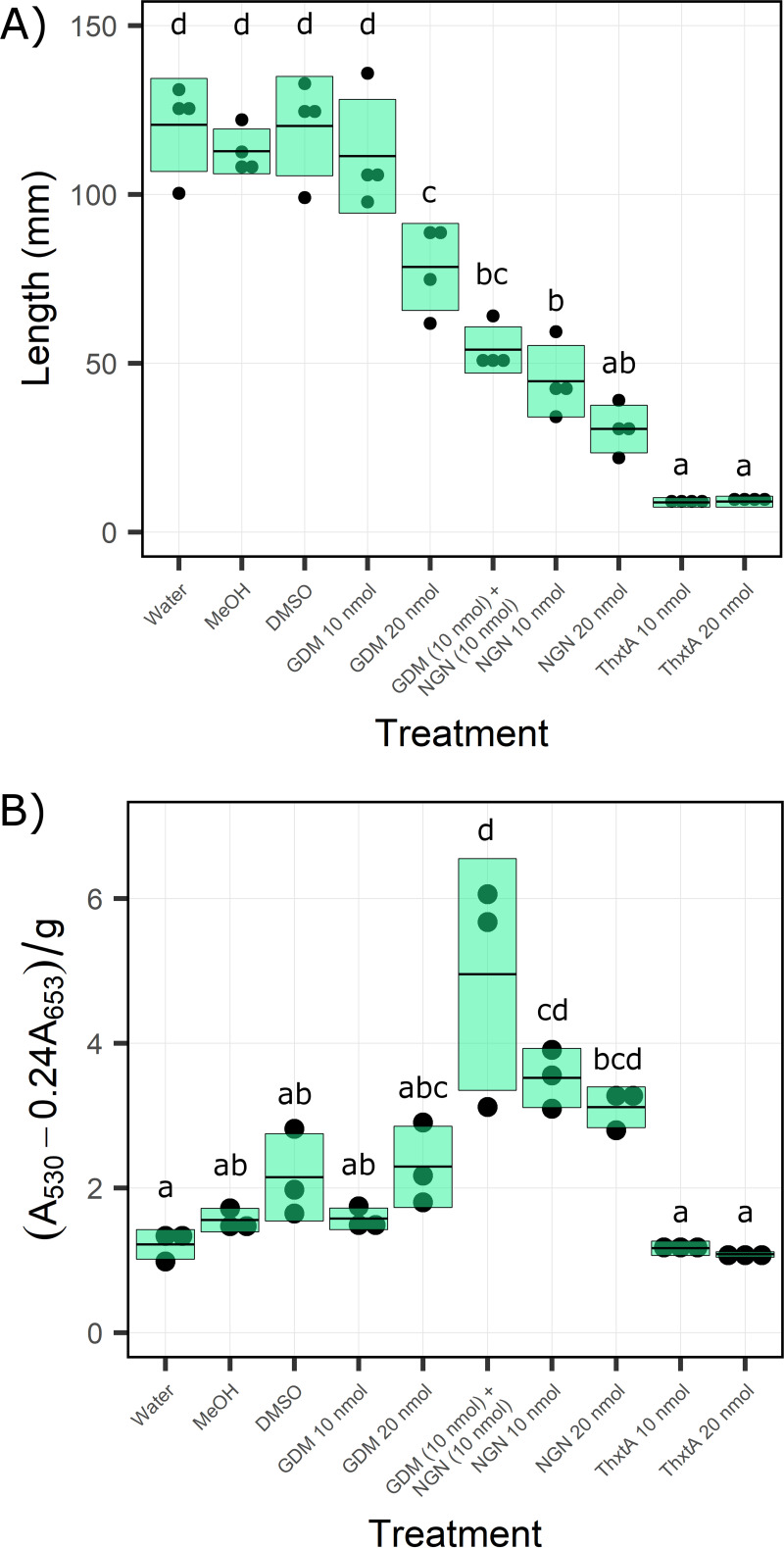
(A) Phytotoxic effect of pure nigericin (NGN), geldanamycin (GDM), and thaxtomin A (ThxtA) on the growth of radish seedlings. Seedlings were treated with 10 or 20 nmol of each compound, or a combination of 10 nmol of each compound. Each box represents the average length of four seedlings plus or minus one standard deviation. (B) Relative quantification of anthocyanin accumulation in radish seedlings treated with nigericin, geldanamycin, and thaxtomin A. Seedlings were treated with 10 or 20 nmol of each compound or a combination of 10 nmol of each compound. Each box represents the average anthocyanin amount from three pairs of seedlings plus or minus one standard deviation. For both experiments, the treatments were analyzed using an ANOVA with Tukey’s test. Values with different letter are statistically different (*P < *0.05). Each assay was performed three times with similar results.

Although nigericin and geldanamycin have been reported to exhibit herbicidal activity against different plants, their phytotoxic effects on radish seedlings and potato tuber tissue have not previously been studied. Therefore, we evaluated the phytotoxic activity of different concentrations of pure nigericin and geldanamycin in our potato tuber slice and radish seedlings bioassays. A pure standard of thaxtomin A, the main phytotoxin produced by *S. scabiei* and other scab-causing pathogens, was included as a positive control in the bioassays. As shown in Fig. 9, the pure geldanamycin caused shallow necrosis of the potato tuber tissue, and the severity of tissue damage increased with increasing amounts of the compound. On the other hand, nigericin did not present significant necrosis like geldanamycin; instead, the inoculation sites showed pitting of the tissue. This effect was also more pronounced with higher amounts of the compound. To determine if the two compounds have a synergistic effect, we treated the tuber tissue with equimolar amounts of both compounds. This resulted in both pitting and necrosis of the tissue that were more severe than those that occurred with treatment with the individual compounds. Notably, the effects of geldanamycin and nigericin were distinct from those of thaxtomin A, which caused dark brown necrosis of the potato tissue without pitting (Fig. 9). Furthermore, the effects of the pure compounds seem relatively less severe than those of the agar cores but more similar to the organic extracts. In the radish seedling bioassay, nigericin had a more significant impact on the growth of the seedlings than geldanamycin at the same concentration (Fig. 10A). The combination treatment (10 nmol of each compound) caused seedling stunting like that caused by the 10 nmol amount of nigericin alone, suggesting that nigericin is primarily responsible for the observed effect (Fig. 10A). This result is in contrast to a previous study that reported that geldanamycin and nigericin have additive effects on the radicle length of different plant species, though this study did not test the effects of these compounds on radish seedlings (49). Notably, neither compound could cause the same degree of seedling stunting as similar amounts of thaxtomin A, indicating that they are less toxic than thaxtomin A against radish seedlings.

The appearance of the seedlings treated with the pure nigericin was notably distinct from that of seedlings subjected to the other treatments. The seedlings exhibited areas of distinct red pigmentation, especially along the edges of the cotyledons. As red pigmentation is an indicator of anthocyanin accumulation (77), we evaluated the levels of anthocyanins in the seedlings subjected to the different treatments. Interestingly, only nigericin caused a significant increase in the level of anthocyanins in the radish seedlings at both test concentrations, while geldanamycin and thaxtomin A did not have a significant effect (Fig. 10B). Treatment with both nigericin and geldanamycin caused the accumulation of an even greater number of anthocyanins, though the level was not significantly different from that resulting from treatment with nigericin alone. To the best of our knowledge, this is the first report of nigericin affecting anthocyanin accumulation in plants, and further work will be required to determine how nigericin and anthocyanin accumulation are connected. Overall, our results demonstrate both that nigericin and geldanamycin display toxic effects against potato tuber tissue and radish seedlings in low amounts and that their effects are distinct from those of thaxtomin A.

### Conclusions.

This study demonstrated for the first time that nigericin and geldanamycin are biosynthesized by the plant pathogen *Streptomyces* sp. 11-1-2, which is closely related to strains within the *S. violaceusniger* subclade. The production of these metabolites was shown to be influenced by the amino sugar NAG, and both compounds were phytotoxic at low quantities against both potato tubers and radish seedlings. Other related compounds were also predicted to be produced by the 11-1-2 strain under the culturing conditions used. The biosynthetic pathways for nigericin and geldanamycin are rich in intermediates and naturally occurring analogues that have been shown to retain or enhance the biological activity of the final products (28, 78). Under this context, we hypothesize that the spectrum of molecules closely related to nigericin and geldanamycin play a role in enhancing the pathogenicity of 11-1-2. Geldanamycin is a benzoquinone ansamycin that acts as an inhibitor of the heat shock protein 90 (HSP90) chaperone (79), and nigericin is a polyether ionophore that facilitates the transport of different ions across membranes (80). Importantly, HSP90 plays a critical role in different plant mechanisms, including resistance to diseases and abiotic disorders (81), and this protein is known to serve as a target for the HopBF1 virulence effector produced by the plant pathogen Pseudomonas syringae pv. *syringae* (82). Nigericin has been shown to disrupt different mechanisms in plants, including the uncoupling of photophosphorylation in the presence of K^+^ ions (83, 84), redirecting of vicilin in pea cotyledons (85), inhibition of protein import into the chloroplast (86), and reduction of photosynthesis (87). Thus, these molecules may contribute to the pathogenicity of *Streptomyces* sp. 11-1-2 by affecting different plant targets and processes. Future work will focus on the construction of geldanamycin and nigericin biosynthetic mutants of *Streptomyces* sp. 11-1-2 so that the role of each compound in plant-pathogen interactions can be further assessed. Given that strains of *S. violaceusniger* and *S. hygroscopicus* have been reported by others as potential biological control agents for the management of fungal plant diseases and potato common scab (88–90), our study additionally highlights the need for potential biological control agents to undergo a thorough assessment for the presence of BGCs that could produce highly phytotoxic compounds.

## MATERIALS AND METHODS

### Bacterial strains and cultivation.

*Streptomyces* sp. strain 11-1-2 was previously isolated from a scab-diseased potato in Newfoundland, Canada (26). The strain was revived from glycerol spore stocks stored at −80°C and was routinely cultivated on potato mash agar (PMA) at 28°C. To evaluate specialized metabolite production, 11-1-2 spores were spread-plated onto OBA (57), mMYM (55), YMS (56), and MMM (58), with and without the addition of *N*-acetylglucosamine (NAG; Sigma-Aldrich Canada) to a final concentration of 50 mM. The plates were then incubated at 28°C for 14 days. To investigate the effects of different concentrations of NAG on specialized metabolite production, the 11-1-2 strain was cultured on plates of mMYM and YMS agar media containing NAG at a final concentration of 10, 20, 50, and 100 mM.

### Bioinformatics analysis.

The genome of 11-1-2 was sequenced previously (38). To characterize its phylogenetic placement, we created an automated multilocus species tree by submitting the genome sequence to the autoMLST website (27). Following the *de novo* workflow, a concatenated alignment was built using the Fast alignment mode (MAFFT FFT-NS-2) with 100 genes (Table S5), followed by an IQ-TREE ultrafast bootstrap analysis (1,000 replicates). The alignment was manually edited to include the most relevant plant-pathogenic *Streptomyces* species and visualized using the R package ggtree v2.2.4 (91). To identify specialized metabolite biosynthetic gene clusters in 11-1-2 and other *Streptomyces* species included in the phylogenetic analysis, we analyzed the genome sequences of these strains using antiSMASH 6.0 (39) with the default parameters.

For the large-scale network and phylogenetic analysis of the nigericin and geldanamycin BGCs, a total of 2,136 *Streptomycetales* genomes (as of May 2021) were downloaded from the National Center for Biotechnology Information (NCBI) and were processed using the command-line version of antiSMASH 5.1.2 with the bacterial setting and otherwise default parameters. Sequence similarity networks and phylogenetic relationships for the nigericin and geldanamycin BGCs were generated using the BiG-SCAPE workflow with the default parameters (https://git.wur.nl/medema-group/BiG-SCAPE) (50). Network files were visualized using Cytoscape version 3.8.2 (92), and the BGCs present within each network were retrieved and compared using Clinker with default parameters (93). Clustermap.js was used to visualize the BGC alignment results (93).

### Organic extraction.

We prepared metabolite extracts used for bioassays from whole plate cultures of 11-1-2 by cutting the agar into pieces and transferring the pieces into a clean flask. Ethyl acetate (20 mL) was added to each flask, and the contents were mixed and left to incubate at room temperature overnight. The extracts were then filtered using Whatman #1 filter paper (GE Healthcare Life Sciences) and transferred into clean flasks. The remaining agar pieces were rinsed with 10 mL of fresh ethyl acetate, which was subsequently filtered and combined with the corresponding extract. The solvent was evaporated overnight, and the dried extracts were resuspended in 1 mL of 100% vol/vol LC-grade methanol and were stored at −20°C.

For the metabolomic analysis of 11-1-2, organic extractions were carried out as described above except that the solvent was removed by rotary evaporation, and the dried extracts were redissolved in 1 mL of 100% vol/vol LC-MS-grade methanol. A 500-μL aliquot of each extract was then transferred into a 96-well plate and used for LC-MS/MS analysis.

### Phytotoxic activity assays.

To evaluate the production of phytotoxic compounds, we performed a potato tuber slice bioassay as described before (94) with some modifications. Briefly, whole potatoes were peeled, disinfected in a 15% vol/vol bleach (Chlorox) solution, and then rinsed using sterile distilled water and cut into 1- to 2-cm-thick slices. Then, four slices were placed into each of three sterile glass petri dishes (150 mm diameter) containing filter paper prewetted with sterile water. Agar cores (8 mm diameter) from OBA, mMYM, YMS, and MMM (with or without NAG) plate cultures of 11-1-2 were placed on top of each slice, and cores from noninoculated media were used as controls. When testing organic culture extracts, sterile, 6-mm Whatman filter disks (GE Healthcare Life Sciences) were placed onto the tuber slices, and then 20 μL of each extract (or 100% methanol) was added to the center of the disk. The plates were sealed with parafilm and incubated in the dark at room temperature for 7 to 10 days, and then the slices were photographed. The assay was performed twice.

Organic culture extracts were also evaluated for phytotoxicity using a radish seedling bioassay. Radish seeds (cv. Cherry Belle) were disinfected with 70% vol/vol ethanol for 5 min and then with 15% vol/vol of bleach for 10 min, after which the seeds were rinsed 10 times with sterile water. The seeds were then placed into a petri dish with prewetted sterile filter paper and were incubated in the dark at 22 to 25°C for 24 h. Germinated seeds showing good development were selected and placed into wells of a 6-well tissue culture plate (two seeds per well). Each well contained 5 mL of sterile water, and 5 μL of each culture extract was added to three separate wells, while control wells were treated with 5 μL of 100% vol/vol methanol or sterile water. The tissue culture plates were wrapped with parafilm and were incubated with shaking (100 rpm) at room temperature (21 to 23°C) under a 16-h photoperiod for 5 days. The total seedling length was determined for each treatment, and the outliers (namely, highest and lowest recorded length per treatment) were removed, resulting in four data points per treatment. The assay was performed three times in total.

The radish seedling bioassay and potato tuber slice bioassay were also performed using solutions of pure nigericin sodium salt (dissolved in 100% vol/vol methanol) and geldanamycin (dissolved in 100% vol/vol dimethyl sulfoxide [DMSO]) (Cayman Chemicals, USA). Radish seedlings were treated with 10 and 20 nmol of each compound, while potato tuber tissue was treated with 5, 10, and 20 nmol of each compound. Additionally, a combination treatment was performed in each assay in which the seedlings or tuber tissue were inoculated with both nigericin and geldanamycin (10 nmol of each compound for the radish bioassay; 2.5, 5, and 10 nmol of each compound for the tuber bioassay). As a positive control for the assays, pure thaxtomin A (dissolved in methanol; Sigma-Aldrich, Canada) at the same test concentrations used for the other compounds was included, while water, methanol, and DMSO were used as negative controls. In addition to measuring the total radish seedling length, the anthocyanin content of the seedlings was evaluated as described by Uppalapati and collaborators (95) with some modifications. Briefly, two representative seedlings per replicate were dried using paper towel, weighted, and then transferred into a 2-mL tube. Then, 1 mL of 3M HCl:H_2_O:methanol (1:3:16) was added to each tube, and the tubes were sealed with parafilm and covered with foil. The tubes were incubated at 15°C and 110 rpm for 24 h, after which the solutions were transferred into fresh tubes. An aliquot of 200 μL of each replicate was transferred into a well of a 96-well plate, and absorbances at 530 and 653 nm (*A*_530_ and *A*_653_) were measured using a Synergy H1 hybrid reader (BIOTEK, Winooski, VT, USA). The anthocyanin content was calculated for each extract using the formula (*A*_530_ – 0.24*A*_653_)/fresh weight (g). The assay was performed twice.

### LC-MS/MS analysis and molecular networking.

LC-MS/MS analysis of culture extracts was performed as described before (55) using a Thermo Fisher Scientific Vanquish ultra high performance LC system coupled to a Thermo Q Exactive Hybrid Quadrupole-Orbitrap mass spectrometer. Metabolite separation was carried out using a Scherzo SM-C_18_ column (2 by 250 mm, 3 μm, 130 Å; Imtakt, United States) maintained at 40°C and utilizing a mobile phase gradient of water/acetonitrile with 0.1% vol/vol formic acid. Mass spectra were recorded in mixed mode following the MS settings. Then, the raw LC-MS/MS data files were converted into mzXML format using MSConvert for further analysis. Both the raw and the converted files are available in the Mass Spectrometry Interactive Virtual Environment (MassIVE) data repository (https://massive.ucsd.edu/ProteoSAFe/static/massive.jsp) under the accession number MSV000086628.

The spectral data obtained were analyzed using the IIMN complement of FBMN (60, 61, 62). For this, the mzXML files were imported and analyzed using MZmine (version 2.37.corr17.7). The parameters used for the analysis are detailed in Table S6. The peak area of each ion in the feature quantification table was adjusted by subtracting the area of the corresponding control medium. The spectral summary files (.mgf files), edited feature quantification tables (.csv files), and supplementary edge files (.csv files) were then processed using the FBMN workflow (61) within the GNPS web platform (https://gnps.ucsd.edu) (62, 96). The parameters of the FBMN analysis are detailed in Table S7. The networks generated were visualized using Cytoscape (92). To further characterize the results in each network, the spectra of compounds without matches to the GNPS reference libraries were analyzed using MetFrag (65) and SIRIUS, including the CSI:fingerID option (63, 64).

### RP-HPLC and LC-MS analysis of organic extracts.

Detection of geldanamycin in culture extracts was completed by reverse-phase high performance liquid chromatography (RP-HPLC) using an Agilent 1260 Infinity Quaternary LC system (Agilent Technologies Canada Inc., Mississauga, ON). Extracts (5 μL) prepared from triplicate cultures were loaded onto a Poroshell 120 EC-C_18_ column (4.6 by 50 mm, 2.7 μm particle size; Agilent Technologies Canada Inc.) held at 40°C. Metabolites were eluted using a linear gradient of acetonitrile and water, each containing 0.1% vol/vol formic acid. The initial mobile phase consisted of 90% water/10% acetonitrile, and this was held constant for 0.2 min before increasing to 0% water/100% acetonitrile over a period of 5.8 min. The mobile phase was maintained at this concentration for 0.4 min and was then returned to 90% water/10% acetonitrile over 0.6 min. The flow rate was held constant at 1 mL/min. Geldanamycin was monitored using a detection wavelength of 308 nm, and the ChemStation software version B.04.03 (Agilent Technologies Canada Inc.) was used for data acquisition. A standard curve was generated using known amounts of a pure geldanamycin standard (Cayman Chemicals, USA) and was used for metabolite quantification. To confirm the presence of geldanamycin, LC-MS analysis of mMYM and YMS (with or without 50 mM NAG) culture extracts was performed using an Agilent 1260 Infinity LC-6230 TOF LC-MS system (Agilent Technologies Canada Inc.) with the same column and separation method as that described above. Mass spectra were recorded in negative mode between 100 and 3,200 *m/z*. Data acquisition was performed using Agilent MassHunter version B.08.00 (Agilent Technologies Canada Inc.), and MestReNova version 14.1.2 (Mestrelab Research S.L.) was used for data analysis.

Nigericin was detected using a modified version of the protocol by Harvey et al. (68). Culture extracts were analyzed using an Agilent 1260 Infinity LC-6230 TOF LC-MS system. Extracts (5 μL) from triplicate cultures were loaded onto a Poroshell 120 EC-C_18_ column (4.6 by 50 mm, 2.7 μm particle size) held at 22°C. The column was equilibrated in 12% 20 mM ammonium acetate buffer/88% methanol, and compounds were eluted using a linear gradient to 100% methanol over 17 min at a constant flow rate of 1 mL/min. Mass spectra were recorded in positive mode between 100 and 3,200 *m/z*. Data acquisition was performed using Agilent MassHunter version B.08.00 (Agilent Technologies Canada Inc.), and MestReNova version 14.1.2 (Mestrelab Research S.L.) was used for data analysis. Quantification of nigericin was achieved by generating a standard curve using known amounts of a pure nigericin sodium salt standard (Cayman Chemicals, USA).

### Statistical analyses.

The results of the radish seedling bioassays with the organic culture extracts were analyzed using the Student’s *t* test function in Microsoft Excel 365. The effects of the culture extracts from the media containing NAG were compared to those of the extracts from the corresponding media lacking NAG, while the water and methanol controls were paired together. For the radish seedling bioassays with the pure compounds, as well as the anthocyanin accumulation assays, the data were analyzed with an analysis of variance and Tukey’s test using the R package “agricolae” and visualized using “ggplot2” and “ggsignif” (97–100). The peak areas of geldanamycin and nigericin, as well as related molecules 15-hydroxygeldanamycin and abierixin, were transformed using the log_10_ function and analyzed using an analysis of variance (ANOVA) paired with Dunnett’s test in Minitab version 20.4. The results were visualized using R packages “ggplot2,” “ggsignif,” and “patchwork” (99–101).
