# Supplementary material for: Nigericin and Geldanamycin Are Phytotoxic Specialized Metabolites Produced by the Plant Pathogen Streptomyces sp. 11-1-2
Source: Microbiol Spectr. 2022 Feb 28;10(2):e02314-21. doi: 10.1128/spectrum.02314-21 (PMC9045263; doi:10.1128/spectrum.02314-21)
Supplement: SUPPLEMENTAL FILE 1 — Supplemental material. Download SPECTRUM02314-21_Supp_1_seq14.pdf, PDF file, 1.1 MB [file spectrum02314-21_supp_1_seq14.pdf]

**Table S1.** Average nucleotide identity (ANI) relative to *Streptomyces* sp. 11-1-2

| Species name | Estimated ANI |
|---|---|
| Streptomyces_hygroscopicus | 0.9866 |
| Streptomyces_violaceusniger_Tu_4113 | 0.9626 |
| Streptomyces_melanosporofaciens | 0.9573 |
| Streptomyces_violaceusniger | 0.9538 |
| Streptomyces_autolyticus | 0.9304 |
| Streptomyces_sp._PRh5 | 0.9297 |
| Streptomyces_iranensis | 0.928 |
| Streptomyces_antioxidans | 0.9215 |
| Streptomyces_sp._NBRC_109436 | 0.9143 |
| Streptomyces_hygroscopicus_subsp._hygroscopicus | 0.9141 |
| Streptomyces_hygroscopicus_subsp._hygroscopicus | 0.9141 |
| Streptomyces_sp._NBRC_110028 | 0.8756 |
| Streptomyces_himastatinicus_ATCC_53653 | 0.8738 |
| Streptomyces_sp._RTd22 | 0.8734 |
| Streptomyces_bingchenggensis_BCW-1 | 0.8583 |
| Streptomyces_sparsogenes | 0.8548 |
| Streptomyces_sclerotialus | 0.821 |
| Streptomyces_violens | 0.821 |
| Streptomyces_niger | 0.8206 |
| Streptomyces_ochraceiscleroticus | 0.8194 |
| Streptomyces_lydicus | 0.8186 |
| Streptomyces_catenulae | 0.8182 |
| Streptomyces_celluloflavus | 0.8173 |
| Streptomyces_rimosus_subsp._rimosus_ATCC_10970 | 0.8173 |
| Streptomyces_varsoviensis | 0.8173 |
| Streptomyces_platensis | 0.8151 |
| Streptomyces_decoyicus | 0.8142 |
| Streptomyces_bungoensis | 0.8132 |
| Streptomyces_yokosukanensis | 0.8132 |
| Streptomyces_leeuwenhoekii | 0.8128 |
| Streptomyces_chattanoogensis | 0.8118 |
| Streptomyces_corchorusii | 0.8113 |
| Streptomyces_ghanaensis_ATCC_14672 | 0.8108 |
| Streptomyces_caatingaensis | 0.8098 |
| Streptomyces_luteus | 0.8093 |
| Streptomyces_noursei_ATCC_11455 | 0.8093 |
| Streptomyces_alboflavus | 0.8088 |
| Streptomyces_ambofaciens_ATCC_23877 | 0.8088 |
| Streptomyces_fodineus | 0.805 |
| Streptomyces_stelliscabiei | 0.8039 |
| Streptomyces_bottropensis_ATCC_25435 | 0.7997 |
| Streptomyces_scabiei_87.22 | 0.7997 |
| Streptomyces_europaeiscabiei | 0.7971 |
| Streptomyces_turgidiscabies | 0.7891 |
| Streptomyces_acidiscabies_84-104 | 0.7841 |
| Nocardiopsis_dassonvillei_subsp._dassonvillei_DSM_43111 | 0.72 |

**Table S2.** Biosynthetic gene clusters predicted in the genome of *Streptomyces* sp. 11-1-2 using antiSMASH 6.0

| Region | Type | From | To | Most similar known cluster | | Similarity |
|---|---|---|---|---|---|---|
| 1 | lanthipeptide-class-i | 244,467 | 267,372 | mycotrienin I | NRP + Polyketide | 7% |
| 2 | terpene | 304,965 | 327,957 | isorenieratene | Terpene | 85% |
| 3 | arylpolyene,ladderane,NRPS | 457,599 | 529,895 | RP-1776 | Polyketide + NRP:Cyclic depsipeptide | 22% |
| 4 | NRPS-like | 571,719 | 614,054 | echoside A / B / C / D / E | NRP | 11% |
| 5 | PKS-like,terpene | 658,010 | 705,793 | rustmicin | Polyketide:Iterative type I | 20% |
| 6 | terpene | 824,320 | 845,259 | tiancilactone | Terpene | 17% |
| 7 | NRPS | 884,096 | 962,151 | dechlorocuracomycin | NRP | 8% |
| 8 | RiPP-like,T1PKS,hglE-KS | 974,268 | 1,097,025 | A83543A | Polyketide | 21% |
| 9 | NRPS-like | 1,160,316 | 1,200,586 | herboxidiene | Polyketide | 3% |
| 10 | T1PKS,NRPS | 1,277,766 | 1,393,079 | meridamycin | NRP + Polyketide | 63% |
| 11 | NAPAA | 1,541,803 | 1,572,879 | paulomycin | Other | 7% |
| 12 | T1PKS | 1,575,870 | 1,705,961 | nigericin | Polyketide:Modular type I | 100% |
| 13 | T1PKS | 1,709,681 | 1,754,690 | salinomycin | Polyketide:Modular type I | 8% |
| 14 | T1PKS,hglE-KS | 2,077,385 | 2,128,172 | asukamycin | Polyketide:Type II | 11% |
| 15 | T1PKS,NRPS-like | 2,322,627 | 2,402,967 | naphthomycin A | Polyketide | 50% |
| 16 | NRPS,nucleoside | 2,436,317 | 2,481,506 | toyocamycin | Other | 30% |
| 17 | NRPS | 2,541,367 | 2,594,739 | glycinocin A | NRP | 11% |
| 18 | terpene | 2,943,967 | 2,964,259 | BE-43547A1 / BE-43547A2 / BE-43547B1 / BE-43547B2 / BE-43547B3 / BE-43547C1 / BE-43547C2 | NRP:Cyclic depsipeptide + Polyketide:Modular type I | 20% |
| 19 | T1PKS,siderophore | 2,991,613 | 3,045,502 | apoptolidin | Polyketide | 25% |
| 20 | ectoine | 3,210,416 | 3,220,820 | ectoine | Other | 100% |
| 21 | terpene | 3,719,051 | 3,738,277 | | | |
| 22 | lanthipeptide-class-i | 3,920,406 | 3,943,726 | | | |
| 23 | T1PKS | 4,304,490 | 4,484,508 | mediomycin A | Polyketide | 68% |
| 24 | RRE-containing | 4,623,525 | 4,643,578 | granaticin | Polyketide:Type II | 10% |
| 25 | ladderane | 4,658,207 | 4,697,921 | atratumycin | NRP | 31% |
| 26 | NRPS | 4,753,653 | 4,795,917 | ochronotic pigment | Other | 50% |
| 27 | indole | 5,011,258 | 5,032,403 | 5-isoprenylindole-3-carboxylate β-D-glycosyl ester | Other | 61% |
| 28 | terpene | 5,596,461 | 5,615,957 | geosmin | Terpene | 100% |
| 29 | siderophore | 6,759,041 | 6,769,871 | desferrioxamin B | Other | 100% |
| 30 | NRPS-like | 7,294,519 | 7,335,806 | echoside A / B / C / D / E | NRP | 100% |
| 31 | siderophore | 7,921,967 | 7,933,880 | | | |
| 32 | ladderane,arylpolyene,NRPS,aminocoumarin | 7,996,321 | 8,069,356 | atratumycin | NRP | 57% |
| 33 | RiPP-like | 8,166,050 | 8,177,396 | | | |
| 34 | T1PKS | 8,239,229 | 8,295,520 | totopotensamide A / B | NRP + Polyketide | 5% |
| 35 | T2PKS | 8,421,342 | 8,493,857 | spore pigment | Polyketide | 83% |
| 36 | terpene | 8,870,148 | 8,892,706 | hopene | Terpene | 76% |

**Table S2.** Biosynthetic gene clusters predicted in the genome of *Streptomyces* sp. 11-1-2 using antiSMASH 6.0

| Region | Type | From | To | Most similar known cluster | | Similarity |
|---|---|---|---|---|---|---|
| 37 | lanthipeptide-class-i | 9,096,140 | 9,120,593 | steffimycin D | Polyketide:Type II + Saccharide:Hybrid/tailoring | 16% |
| 38 | terpene | 9,210,479 | 9,230,310 | lasalocid | Polyketide | 3% |
| 39 | T1PKS | 9,358,088 | 9,405,558 | | | |
| 40 | NRPS,transAT-PKS,NRPS-like | 9,419,881 | 9,495,386 | meilingmycin | Polyketide | 7% |
| 41 | butyrolactone | 9,654,449 | 9,665,381 | | | |
| 42 | hserlactone | 9,675,690 | 9,696,448 | daptomycin | NRP | 3% |
| 43 | redox-cofactor | 9,815,666 | 9,837,784 | lankacidin C | NRP + Polyketide | 13% |
| 44 | T1PKS | 9,877,490 | 9,945,918 | elaiophylin | Polyketide | 87% |
| 45 | T1PKS,NRPS-like | 10,159,131 | 10,240,039 | Geldanamycin | Polyketide | 100% |
| 46 | terpene | 10,383,924 | 10,403,386 | pristinol | Terpene | 100% |
| 47 | terpene | 10,508,385 | 10,529,722 | ebelactone | Polyketide | 8% |
| 48 | T1PKS | 10,696,374 | 10,842,668 | niphimycins C-E | Polyketide | 87% |
| 49 | NAPAA | 10,939,213 | 10,973,346 | | | |
| 50 | NRPS,T1PKS | 11,092,881 | 11,205,821 | coelichelin | NRP | 90% |
| 51 | betalactone | 11,367,403 | 11,392,789 | | | |

**Table S3.** Genome accessions used for phylogenetic tree and antiSMASH analysis

| Species name | Accession |
|---|---|
| Nocardiopsis dassonvillei subsp. dassonvillei DSM 43111 | CP002040.1 |
| Streptomyces alboflavus NRRL B-2373 | JNXT00000000.1 |
| Streptomyces ambofaciens ATCC 23877 | CP012382.1 |
| Streptomyces antioxidans MUSC 164 | LAKD00000000 |
| Streptomyces autolyticus CGMCC0516 | CP019458 |
| Streptomyces bottropensis ATCC 25435 | ARTP00000000 |
| Streptomyces bungoensis DSM 41781 | LMWX00000000 |
| Streptomyces caatingaensis CMAA-1322 | LFXA00000000 |
| Streptomyces catenulae NRRL B-2342 | JODY00000000.1 |
| Streptomyces celluloflavus NRRL B-2493 | JOEL00000000 |
| Streptomyces chattanoogensis NRRL ISP-5002 | LGKG00000000 |
| Streptomyces corchorusii DSM 40340 | LMWP00000000 |
| Streptomyces decoyicus NRRL 2666 | LGUU00000000 |
| Streptomyces europaeiscabiei NCPPB-4064 | JPPV00000000 |
| Streptomyces fodineus TW1S1 | CP017248 |
| Streptomyces himastatinicus ATCC 53653 | ACEX00000000.1 |
| Streptomyces hygroscopicus subsp. hygroscopicus NBRC 100766 | BCAN00000000 |
| Streptomyces hygroscopicus subsp. hygroscopicus NBRC 16556 | BBOU00000000 |
| Streptomyces hygroscopicus subsp. hygroscopicus NRRL B-1477 | JOIK00000000.1 |
| Streptomyces hygroscopicus subsp. hygroscopicus strain OsiSh-1 | MDFG00000000 |
| Streptomyces hygroscopicus XM201 | CP018627 |
| Streptomyces iranensis | LK022848 |
| Streptomyces leeuwenhoekii C34 DSM 42122 NRRL B-24963 | LN831790.1 |
| Streptomyces luteus TRM 45540 | JNFQ00000000 |
| Streptomyces lydicus NRRL ISP-5461 | JNZA00000000.1 |
| Streptomyces melanosporofaciens DSM 40318 | FNST00000000 |
| Streptomyces niger NRRL B-3857 | JOFQ00000000 |
| Streptomyces noursei ATCC 11455 | CP011533.1 |
| Streptomyces ochraceiscleroticus NRRL ISP-5594 | JOAX00000000.1 |
| Streptomyces platensis DSM 40041 | MIGA00000000 |
| Streptomyces rimosus subsp. rimosus ATCC 10970 | CP023688.1 |
| Streptomyces rimosus subsp. rimosus NRRL ISP-5260 | JNYR00000000 |
| Streptomyces sp. 11-1-2 | CP022545 |
| Streptomyces sp. NBRC 109436 | BBON00000000.1 |
| Streptomyces sp. NBRC 110028 | BBUZ00000000.1 |
| Streptomyces sp. PRh5 | JABQ01000001 |
| Streptomyces sp. SPMA113 | BDFA00000000.1 |
| Streptomyces sparsogenes ATCC 25498 | MAXF00000000.1 |
| Streptomyces stelliscabiei strain P3825 | JPPZ00000000 |
| Streptomyces turgidiscabies T45 | BCMN00000000 |
| Streptomyces varsoviensis NRRL B-3589 | JOFN00000000 |
| Streptomyces violaceusniger NRLL F-8817 | LLZJ00000000 |
| Streptomyces violaceusniger Tu 1443 | CP002994 |
| Streptomyces violens NRRL ISP-5597 | JOBH00000000 |
| Streptomyces viridosporus ATCC 14672 | ABYA00000000 |
| Streptomyces yokosukanensis DSM 40224 | LMWN00000000 |
| Streptomyces sp. RTd22 | CP015726 |
| Streptomyces bingchenggensis BCW-1 | NC_016582 |
| Streptomyces scabiei 87-22 | NC_013929 |
| Streptomyces acidiscabies 84-104 | AHBF01000001.1 |

**Table S4**. DasR binding sites predicted in the genome of *Streptomyces* sp. 11-1-2 as calculated by PREDetector

| Gene locus | Gene product | Sequence | Position | Score | Region | Co-transcribed locus | Co-transcribed gene product | Region name |
|---|---|---|---|---|---|---|---|---|
| CGL27_RS07365 [CGL27_07235] | chitinase | ACTGGTCTAGTCCTGT | -112 | 16.6 | regulatory | | | UPS1216 |
| CGL27_RS22220 [CGL27_21915] | ROK family protein | AGTGGACTAGACCTCT | -44 | 16.5 | regulatory | CGL27_RS22225 [CGL27_21920] (nagA) | N-acetylglucosamine-6-phosphate deacetylase | UPS3746 |
| CGL27_RS22215 [CGL27_21910] | extracellular solute-binding protein | AGTGGACTAGACCTCT | -264 | 16.5 | regulatory | | | UPS3746 |
| CGL27_RS31315 [CGL27_30985] | ATPase | TCTGGACTAGACCACT | -85 | 16.1 | regulatory | | | UPS5280 |
| CGL27_RS19705 [CGL27_19420] | PTS lactose transporter subunit IIC | ACAGGTCTACACCACA | -80 | 15.5 | regulatory | | | UPS3309 |
| CGL27_RS19700 [CGL27_19415] | PTS sugar transporter | ACAGGTCTACACCACA | -146 | 15.5 | regulatory | | | UPS3309 |
| CGL27_RS25640 [CGL27_25340] | tRNA-Gly | AGAGGTCTAGACAACA | 36 | 15 | terminator | | | UPS4324 |
| CGL27_RS25635 [CGL27_25335] | site-specific integrase | AGAGGTCTAGACAACA | 1 | 15 | terminator | | | UPS4324 |
| CGL27_RS19705 [CGL27_19420] | PTS lactose transporter subunit IIC | AATGGTCTACACCATT | -163 | 14.9 | regulatory | | | UPS3309 |
| CGL27_RS19700 [CGL27_19415] | PTS sugar transporter | AATGGTCTACACCATT | -63 | 14.9 | regulatory | | | UPS3309 |
| CGL27_RS17585 [CGL27_17285] (leuA) | 2-isopropylmalate synthase | GGAGGTGTAGACCAGA | 1085 | 14.5 | coding | | | CGL27_RS17585 [CGL27_17285] (leuA) |
| CGL27_RS31290 [CGL27_30960] | extracellular solute-binding protein | ACTGGTCTGGACCATT | -49 | 14.5 | regulatory | CGL27_RS31295 [CGL27_30965]; CGL27_RS31300 [CGL27_30970]; CGL27_RS31305 [CGL27_30975] | sugar ABC transporter permease; carbohydrate ABC transporter permease; glycoside hydrolase family 3 protein | UPS5276 |
| CGL27_RS31285 [CGL27_30955] | GntR family transcriptional regulator | ACTGGTCTGGACCATT | -228 | 14.5 | regulatory | | | UPS5276 |

**Table S4**. DasR binding sites predicted in the genome of *Streptomyces* sp. 11-1-2 as calculated by PREDetector

| Gene locus | Gene product | Sequence | Position | Score | Region | Co-transcribed locus | Co-transcribed gene product | Region name |
|---|---|---|---|---|---|---|---|---|
| CGL27_RS39880 [CGL27_39435] | acyl-CoA dehydrogenase | GAAGGACTAGACCAGT | -290 | 13.7 | regulatory | | | UPS6714 |
| CGL27_RS39875 [CGL27_39430] | glycoside hydrolase family 18 protein | GAAGGACTAGACCAGT | -111 | 13.7 | regulatory | | | UPS6714 |
| CGL27_RS04780 [CGL27_04740] | sugar hydrolase | ACTGGTCTAGACATAC | -113 | 13.3 | regulatory | CGL27_RS04775 [CGL27_04735] | glycosyl hydrolase | UPS0793 |
| CGL27_RS04785 [CGL27_04745] | TetR/AcrR family transcriptional regulator | ACTGGTCTAGACATAC | -446 | 13.3 | upstream | | | UPS0793 |
| CGL27_RS36175 [CGL27_35785] | peptide ABC transporter substrate-binding protein | AGTGGACTGGACCAGT | -65 | 13.2 | regulatory | CGL27_RS36180 [CGL27_35790]; CGL27_RS36185 [CGL27_35795]; CGL27_RS36190 [CGL27_35800]; CGL27_RS36195 [CGL27_35805] | M20/M25/M40 family metallo-hydrolase; S9 family peptidase; 4'-phosphopantetheinyl transferase superfamily protein; N-acetyltransferase | UPS6119 |
| CGL27_RS18555 [CGL27_18270] | sugar ABC transporter substrate-binding protein | TCAGGTCTAGACGTGT | -213 | 12.9 | regulatory | CGL27_RS18560 [CGL27_18275]; CGL27_RS18565 [CGL27_18280] | ABC transporter permease; sugar ABC transporter ATP-binding protein | UPS3111 |
| CGL27_RS18550 [CGL27_18265] (iolC) | 5-dehydro-2-deoxygluconokinase | TCAGGTCTAGACGTGT | -84 | 12.9 | regulatory | | | UPS3111 |
| CGL27_RS34965 [CGL27_34620] (ngcE) | carbohydrate ABC transporter N-acetylglucosamine/diacetylchitobiose-binding protein | AGTGGACTATACCTGT | -209 | 12.9 | regulatory | | | UPS5911 |
| CGL27_RS34960 [CGL27_34615] | hypothetical protein | AGTGGACTATACCTGT | -106 | 12.9 | regulatory | | | UPS5911 |
| CGL27_RS12990 [CGL27_12765] | CTP synthase | GGTGGAGTAGACCTGT | 278 | 12.7 | coding | | | CGL27_RS12990 [CGL27_12765] |

**Table S4**. DasR binding sites predicted in the genome of *Streptomyces* sp. 11-1-2 as calculated by PREDetector

| Gene locus | Gene product | Sequence | Position | Score | Region | Co-transcribed locus | Co-transcribed gene product | Region name |
|---|---|---|---|---|---|---|---|---|
| CGL27_RS34310 [CGL27_33980] | GNAT family N-acetyltransferase | TGTTGTCTAGACCAAA | -162 | 12.7 | regulatory | | | UPS5814 |
| CGL27_RS34305 [CGL27_33975] | HPr family phosphocarrier protein | TGTTGTCTAGACCAAA | -44 | 12.7 | regulatory | | | UPS5814 |
| CGL27_RS47585 [CGL27_47080] | alpha-mannosidase | ACTGGTCCACACCACA | 314 | 12.6 | coding | | | CGL27_RS47585 [CGL27_47080] |
| CGL27_RS23245 [CGL27_22930] (acs) | acetate--CoA ligase | ACAGGTCTAAACCAAT | -103 | 12.4 | regulatory | | | UPS3916 |
| CGL27_RS32530 [CGL27_32195] | DinB family protein | ACTGGACTCGACCTGT | -110 | 12.4 | regulatory | | | UPS5498 |
| CGL27_RS32525 [CGL27_32190] | M4 family peptidase | ACTGGACTCGACCTGT | -285 | 12.4 | regulatory | | | UPS5498 |
| CGL27_RS25565 [CGL27_25265] | ATP-binding protein | CGTGGTCTAGACAACA | -2468 | 12.3 | upstream | | | UPS4312 |
| CGL27_RS30200 [CGL27_29870] | hypothetical protein | AGTGGTCCAGACCAAT | -202 | 12.3 | regulatory | CGL27_RS30205 [CGL27_29875] (cpaB); CGL27_RS30210 [CGL27_29880] | Flp pilus assembly protein CpaB; ParA family protein | UPS5088 |
| CGL27_RS30195 [CGL27_29865] | chitinase | AGTGGTCCAGACCAAT | -95 | 12.3 | regulatory | | | UPS5088 |
| CGL27_RS21320 [CGL27_21025] | hypothetical protein | ACTGGTCCAGACCTGC | -98 | 11.8 | regulatory | | | UPS3585 |
| CGL27_RS33065 [CGL27_32735] | tRNA-Gln | ACTGGTCTATACCATG | -14 | 11.7 | regulatory | | | CGL27_RS33065 [CGL27_32735] |
| CGL27_RS33060 [CGL27_32730] | tRNA-Glu | ACTGGTCTATACCATG | 72 | 11.7 | coding | | | CGL27_RS33065 [CGL27_32735] |
| CGL27_RS21150 [CGL27_20855] | alpha/beta hydrolase | AGTGGAGTACACCAAT | -36 | 11.7 | regulatory | | | UPS3560 |
| CGL27_RS21145 [CGL27_20850] | adenosine deaminase | AGTGGAGTACACCAAT | -83 | 11.7 | regulatory | | | UPS3560 |
| CGL27_RS39605 [CGL27_39175] | FAD-dependent oxidoreductase | ACTGGTCTGCACCTCC | 773 | 11.6 | coding | | | CGL27_RS39605 [CGL27_39175] |
| CGL27_RS45830 [CGL27_45345] | hypothetical protein | ACAGGTCTACACGACC | 1700 | 11.6 | coding | | | CGL27_RS45830 [CGL27_45345] |
| CGL27_RS08795 [CGL27_08645] | sugar hydrolase | CCAGGTCTAGACCAAT | -194 | 11.6 | regulatory | | | UPS1449 |

**Table S4**. DasR binding sites predicted in the genome of *Streptomyces* sp. 11-1-2 as calculated by PREDetector

| Gene locus | Gene product | Sequence | Position | Score | Region | Co-transcribed locus | Co-transcribed gene product | Region name |
|---|---|---|---|---|---|---|---|---|
| CGL27_RS25590 [CGL27_25290] | hypothetical protein | ACTCATCTACACAACT | -48 | 11.5 | regulatory | CGL27_RS25595 [CGL27_25295] | conjugal transfer protein TraS | UPS4316 |
| CGL27_RS01490 [CGL27_01440] | alpha-L-rhamnosidase | ACTGGTCCATACCTGT | -321 | 11.4 | regulatory | | | UPS0254 |
| CGL27_RS19705 [CGL27_19420] | PTS lactose transporter subunit IIC | TGTGGTTTAGACCATA | -64 | 11.4 | regulatory | | | UPS3309 |
| CGL27_RS19700 [CGL27_19415] | PTS sugar transporter | TGTGGTTTAGACCATA | -162 | 11.4 | regulatory | | | UPS3309 |
| CGL27_RS33775 [CGL27_33445] (eccCa) | type VII secretion protein EccCa | AGTGGTGTCCACAACT | 490 | 11.3 | coding | | | CGL27_RS33775 [CGL27_33445] (eccCa) |
| CGL27_RS22135 [CGL27_21830] | copper homeostasis protein CutC | ATTGGTCTAGACATGG | -2 | 11.2 | regulatory | | | UPS3732 |
| CGL27_RS22300 [CGL27_21995] | DNA-binding response regulator | ACTGGTCGAGACCGGT | -792 | 11.1 | upstream | | | UPS3761 |
| CGL27_RS43500 [CGL27_43040] | chitin-binding protein | AGTGGACCAGACCACA | -201 | 11.1 | regulatory | | | UPS7305 |
| CGL27_RS19970 [CGL27_19680] | extracellular solute-binding protein | AGAGGTCTGAACCACT | -110 | 11 | regulatory | CGL27_RS19965 [CGL27_19675]; CGL27_RS19960 [CGL27_19670] | sugar ABC transporter permease; carbohydrate ABC transporter permease | UPS3352 |
| CGL27_RS31080 [CGL27_30755] | hypothetical protein | AAGGGTGTACTCCACT | -123 | 10.9 | regulatory | | | UPS5239 |
| CGL27_RS26245 [CGL27_25940] | XRE family transcriptional regulator | ACAGGTCGAGACAACC | 480 | 10.8 | coding | CGL27_RS26240 [CGL27_25935] | DUF397 domain-containing protein | CGL27_RS26245 [CGL27_25940] |
| CGL27_RS48995 [CGL27_48460] | FAD-dependent oxidoreductase | ACTGGTGTAAACCAGG | -238 | 10.8 | regulatory | | | UPS8206 |

**Table S5**. Genes used for the construction of the multilocus species tree of *Streptomyces* sp. 11-1-2 in autoMLST

| Accession number | Gene | Function | Description |
|---|---|---|---|
| TIGR00133 | gatB | Protein synthesis | aspartyl/glutamyl-tRNA(Asn/Gln) amidotransferase, B subunit |
| TIGR00132 | gatA | Protein synthesis | aspartyl/glutamyl-tRNA(Asn/Gln) amidotransferase, A subunit |
| TIGR03953 | rplD_bact | Protein synthesis | 50S ribosomal protein uL4 |
| TIGR01959 | nuoF_fam | Energy metabolism | NADH oxidoreductase (quinone), F subunit |
| TIGR00138 | rsmG_gidB | Protein synthesis | 16S rRNA (guanine(527)-N(7))-methyltransferase RsmG |
| TIGR01816 | sdhA_forward | Energy metabolism | succinate dehydrogenase, flavoprotein subunit |
| TIGR01520 | FruBisAldo_II_A | Energy metabolism | fructose-bisphosphate aldolase, class II |
| TIGR00036 | dapB | Amino acid biosynthesis | 4-hydroxy-tetrahydrodipicolinate reductase |
| TIGR01529 | argR_whole | Regulatory functions | arginine repressor |
| TIGR00763 | lon | Protein fate | endopeptidase La |
| TIGR00447 | pth | Protein synthesis | aminoacyl-tRNA hydrolase |
| TIGR02692 | tRNA_CCA_actino | Protein synthesis | CCA tRNA nucleotidyltransferase |
| TIGR00331 | hrcA | Regulatory functions | heat-inducible transcription repressor HrcA |
| TIGR01049 | rpsJ_bact | Protein synthesis | ribosomal protein uS10 |
| TIGR01044 | rplV_bact | Protein synthesis | ribosomal protein uL22 |
| TIGR00338 | serB | Amino acid biosynthesis | phosphoserine phosphatase SerB |
| TIGR00962 | atpA | Energy metabolism | ATP synthase F1, alpha subunit |
| TIGR01127 | ilvA_1Cterm | Amino acid biosynthesis | threonine ammonia-lyase |
| TIGR01966 | RNasePH | Transcription | ribonuclease PH |
| TIGR00459 | aspS_bact | Protein synthesis | aspartate--tRNA ligase |
| TIGR00754 | bfr | Transport and binding proteins | bacterioferritin |
| TIGR02504 | NrdJ_Z | Purines, pyrimidines, nucleosides, and nucleotides | ribonucleoside-diphosphate reductase, adenosylcobalamin-dependent |
| TIGR03594 | GTPase_EngA | Protein synthesis | ribosome-associated GTPase EngA |
| TIGR00088 | trmD | Protein synthesis | tRNA (guanine(37)-N(1))-methyltransferase |
| TIGR00855 | L12 | Protein synthesis | ribosomal protein bL12 |
| TIGR00615 | recR | DNA metabolism | recombination protein RecR |
| TIGR00244 | TIGR00244 | Regulatory functions | transcriptional regulator NrdR |
| TIGR01039 | atpD | Energy metabolism | ATP synthase F1, beta subunit |
| TIGR01134 | purF | Purines, pyrimidines, nucleosides, and nucleotides | amidophosphoribosyltransferase |
| TIGR01137 | cysta_beta | Amino acid biosynthesis | cystathionine beta-synthase |
| TIGR00690 | rpoZ | Transcription | DNA-directed RNA polymerase, omega subunit |
| TIGR01032 | rplT_bact | Protein synthesis | ribosomal protein bL20 |
| TIGR00019 | prfA | Protein synthesis | peptide chain release factor 1 |
| TIGR00012 | L29 | Protein synthesis | ribosomal protein uL29 |
| TIGR01978 | sufC | Biosynthesis of cofactors, prosthetic groups, and carriers | FeS assembly ATPase SufC |
| TIGR02673 | FtsE | Cellular processes | cell division ATP-binding protein FtsE |
| TIGR00150 | T6A_YjeE | Protein synthesis | tRNA threonylcarbamoyl adenosine modification protein YjeE |
| TIGR01389 | recQ | DNA metabolism | ATP-dependent DNA helicase RecQ |
| TIGR00090 | rsfS_iojap_ybeB | Protein synthesis | ribosome silencing factor |
| TIGR00554 | panK_bact | Biosynthesis of cofactors, prosthetic groups, and carriers | pantothenate kinase |

**Table S5**. Genes used for the construction of the multilocus species tree of *Streptomyces* sp. 11-1-2 in autoMLST

| Accession number | Gene | Function | Description |
|---|---|---|---|
| TIGR02127 | pyrF_sub2 | Purines, pyrimidines, nucleosides, and nucleotides | orotidine 5'-phosphate decarboxylase |
| TIGR00096 | TIGR00096 | Protein synthesis | 16S rRNA (cytidine(1402)-2'-O)-methyltransferase |
| TIGR01302 | IMP_dehydrog | Purines, pyrimidines, nucleosides, and nucleotides | inosine-5'-monophosphate dehydrogenase |
| TIGR03800 | PLP_synth_Pdx2 | Biosynthesis of cofactors, prosthetic groups, and carriers | pyridoxal 5'-phosphate synthase, glutaminase subunit Pdx2 |
| TIGR01022 | rpmJ_bact | Protein synthesis | ribosomal protein bL36 |
| TIGR01021 | rpsE_bact | Protein synthesis | ribosomal protein uS5 |
| TIGR00065 | ftsZ | Cellular processes | cell division protein FtsZ |
| TIGR00064 | ftsY | Protein fate | signal recognition particle-docking protein FtsY |
| TIGR00060 | L18_bact | Protein synthesis | ribosomal protein uL18 |
| TIGR00062 | L27 | Protein synthesis | ribosomal protein bL27 |
| TIGR01394 | TypA_BipA | Regulatory functions | GTP-binding protein TypA/BipA |
| TIGR00639 | PurN | Purines, pyrimidines, nucleosides, and nucleotides | phosphoribosylglycinamide formyltransferase |
| TIGR00302 | TIGR00302 | Purines, pyrimidines, nucleosides, and nucleotides | phosphoribosylformylglycinamidine synthase, purS protein |
| TIGR00431 | TruB | Protein synthesis | tRNA pseudouridine(55) synthase |
| TIGR00263 | trpB | Amino acid biosynthesis | tryptophan synthase, beta subunit |
| TIGR01011 | rpsB_bact | Protein synthesis | ribosomal protein uS2 |
| TIGR01855 | IMP_synth_hisH | Amino acid biosynthesis | imidazole glycerol phosphate synthase, glutamine amidotransferase subunit |
| TIGR00409 | proS_fam_II | Protein synthesis | proline--tRNA ligase |
| TIGR03705 | poly_P_kin | Central intermediary metabolism | polyphosphate kinase 1 |
| TIGR01169 | rplA_bact | Protein synthesis | ribosomal protein uL1 |
| TIGR00482 | TIGR00482 | Biosynthesis of cofactors, prosthetic groups, and carriers | nicotinate (nicotinamide) nucleotide adenylyltransferase |
| TIGR01164 | rplP_bact | Protein synthesis | ribosomal protein uL16 |
| TIGR01009 | rpsC_bact | Protein synthesis | ribosomal protein uS3 |
| TIGR00168 | infC | Protein synthesis | translation initiation factor IF-3 |
| TIGR02012 | tigrfam_recA | DNA metabolism | protein RecA |
| TIGR02970 | succ_dehyd_cytB | Energy metabolism | succinate dehydrogenase, cytochrome b556 subunit |
| TIGR00086 | smpB | Protein synthesis | SsrA-binding protein |
| TIGR00048 | rRNA_mod_RlmN | Protein synthesis | 23S rRNA (adenine(2503)-C(2))-methyltransferase |
| TIGR00184 | purA | Purines, pyrimidines, nucleosides, and nucleotides | adenylosuccinate synthase |
| TIGR00166 | S6 | Protein synthesis | ribosomal protein bS6 |
| TIGR02075 | pyrH_bact | Purines, pyrimidines, nucleosides, and nucleotides | UMP kinase |
| TIGR00651 | pta | Energy metabolism | phosphate acetyltransferase |
| TIGR00498 | lexA | Regulatory functions | repressor LexA |

**Table S5**. Genes used for the construction of the multilocus species tree of *Streptomyces* sp. 11-1-2 in autoMLST

| Accession number | Gene | Function | Description |
|---|---|---|---|
| TIGR03654 | L6_bact | Protein synthesis | ribosomal protein uL6 |
| TIGR01171 | rplB_bact | Protein synthesis | ribosomal protein uL2 |
| TIGR00959 | ffh | Protein fate | signal recognition particle protein |
| TIGR01455 | glmM | Central intermediary metabolism | phosphoglucosamine mutase |
| TIGR01071 | rplO_bact | Protein synthesis | ribosomal protein uL15 |
| TIGR01073 | pcrA | DNA metabolism | ATP-dependent DNA helicase PcrA |
| TIGR00952 | S15_bact | Protein synthesis | ribosomal protein uS15 |
| TIGR00700 | GABAtrnsam | Central intermediary metabolism | 4-aminobutyrate transaminase |
| TIGR01632 | L11_bact | Protein synthesis | ribosomal protein uL11 |
| TIGR00461 | gcvP | Energy metabolism | glycine dehydrogenase |
| TIGR00190 | thiC | Biosynthesis of cofactors, prosthetic groups, and carriers | phosphomethylpyrimidine synthase |
| TIGR00468 | pheS | Protein synthesis | phenylalanine--tRNA ligase, alpha subunit |
| TIGR01736 | FGAM_synth_II | Purines, pyrimidines, nucleosides, and nucleotides | phosphoribosylformylglycinamidine synthase II |
| TIGR01737 | FGAM_synth_I | Purines, pyrimidines, nucleosides, and nucleotides | phosphoribosylformylglycinamidine synthase I |
| TIGR01066 | rplM_bact | Protein synthesis | ribosomal protein uL13 |
| TIGR01067 | rplN_bact | Protein synthesis | ribosomal protein uL14 |
| TIGR01063 | gyrA | DNA metabolism | DNA gyrase, A subunit |
| TIGR00580 | mfd | DNA metabolism | transcription-repair coupling factor |
| TIGR00228 | ruvC | DNA metabolism | crossover junction endodeoxyribonuclease RuvC |
| TIGR00670 | asp_carb_tr | Purines, pyrimidines, nucleosides, and nucleotides | aspartate carbamoyltransferase |
| TIGR03635 | uS17_bact | Protein synthesis | ribosomal protein uS17 |
| TIGR01059 | gyrB | DNA metabolism | DNA gyrase, B subunit |
| TIGR00343 | TIGR00343 | Biosynthesis of cofactors, prosthetic groups, and carriers | pyridoxal 5'-phosphate synthase, synthase subunit Pdx1 |
| TIGR03631 | uS13_bact | Protein synthesis | ribosomal protein uS13 |
| TIGR03632 | uS11_bact | Protein synthesis | ribosomal protein uS11 |
| TIGR01051 | topA_bact | DNA metabolism | DNA topoisomerase I |
| TIGR01050 | rpsS_bact | Protein synthesis | ribosomal protein uS19 |

**Table S6.** Parameters used in Mzmine to perform Ion Identity Molecular Networking.

| Processing step | Parameter | Value |
|---|---|---|
| Mass detection MS1 | Scans | 1 |
| | Mass detector | 15000 |
| | Mass list name | masses |
| Mass detection MS2 | Scans | 2 |
| | Mass detector | 1000 |
| | Mass list name | masses |
| Chromatogram builder - ADAP | Scans | 4 |
| | Mass list | masses |
| | Group intensity threshold | 500000 |
| | Min height | 50000 |
| | m/z tolerance | 0.01; 10 ppm |
| | Suffix | chromatograms |
| Chromatogram deconvolution | Suffix | deconvoluted |
| | Algorithm | S/N threshold = 10; min feature height 50000; coefficient/area threshold = 60; peak duration range = 0.01-0.5 min, RT wavelet range = 0.01-0.1 |
| | m/z center calculation | Median |
| | m/z range for MS2 scan pairing (Da) | 0.02 |
| | RT range for MS2 scan pairing (min) | 0.2 |
| Isotopic peaks grouper | Suffix | deisotoped |
| | m/z tolerance | 0.01 ; 10 ppm |
| | Retention time tolerance | 0.5 |
| | Monotonic shape | Checked |
| | Maximum charge | 4 |
| | Representative isotope | Most intense |
| Join Aligner | Peak list name | Aligned peak lists |
| | m/z tolerance | 0.01; 10 ppm |
| | Weight for m/z | 75 |
| | Retention time tolerance | 0.5 |
| | Weight for RT | 25 |
| | Require same charge state | No |
| Peak finder (multithreaded) | Suffix | gap-filled |
| | Intensity Tolerance | 5% |
| | m/z tolerance | 0.01; 5 ppm |
| | Retention time tolerance | 0.15 |

**Table S6.** Parameters used in Mzmine to perform Ion Identity Molecular Networking.

| Processing step | Parameter | Value |
|---|---|---|
| Peak list rows filter | Suffix | filtered |
| | Minimum peaks in a row | 2 |
| | Minimum peaks in an isotope pattern | 2 |
| | Keep or remove rows | Keep all that match criteria |
| | Keep only peaks with MS2 scan | Checked |
| | Reset the peak number ID | Checked |
| Metacorrelate | RT tolerance | 0.1 |
| | Min height | 10000 |
| | Noise level | 1000 |
| | Correlation grouping | Checked; min data points 5, Min data points on edge 2; measure PEARSON; min feature shape correlation 85% |
| | Feature height correlation | Checked; min data points 3, measure PEARSON; min correlation 70% |
| Ion identity networking | m/z tolerance | 0.001; 10 ppm |
| | Check | ONE FEATURE |
| | Min height | 100000 |
| | Annotation refinement | No |
| | Ion identity library | Positive; max charge 2; max mol/cluster 2<br>Adducts: [M+H]+; [M+Na]+; [M+NH4]+; [M+2H]2+; [M+H+Na]2+<br>Modifications: [M-H2O]; [M-2H2O] |
| | Ion identity library | Negative; max charge 2; max mol/cluster 2<br>Adducts: [M-H]-; [M+Cl]-<br>Modifications: [M-H2O]; [M-2H2O] |
| Add Ion identities to network | m/z tolerance | 0.001; 10 ppm |
| | Min height | 100000 |
| | Ion identity library | Adducts: [M+H]+; [M+Na]+; [M+K]+; [M+NH4]+; [M+2H]2+; [M+Ca]2+; [M+Fe]2+; [M+H+Na]2+; [M+H+NH4]2+; [M-H+2Na]; [M+Ca-H]+; [M+Fe-H]+ |
| Add Ion identities to network | m/z tolerance | 0.001; 10 ppm |
| | Min height | 100000 |
| | Ion identity library | Adducts: [M-H]-; [M-2H+Na]-; [M+Cl]-; [M+FA]-<br>Modifications: [M-H2O]; [M-2H2O]; [M-NH3] |

**Table S7.** Global natural products social molecular networking (GNPS) parameters used for analysis.

| Parameter* | | Value |
|---|---|---|
| Basic options | Precursor Ion Mass Tolerance | 0.02 |
| | Fragment Ion Mass Tolerance | 0.02 |
| Advanced Network Options | Min pairs Cos | 0.7 |
| | Min matched fragment ions | 6 |
| | Network TopK | 10 |

*Default values were kept for any other parameter not listed

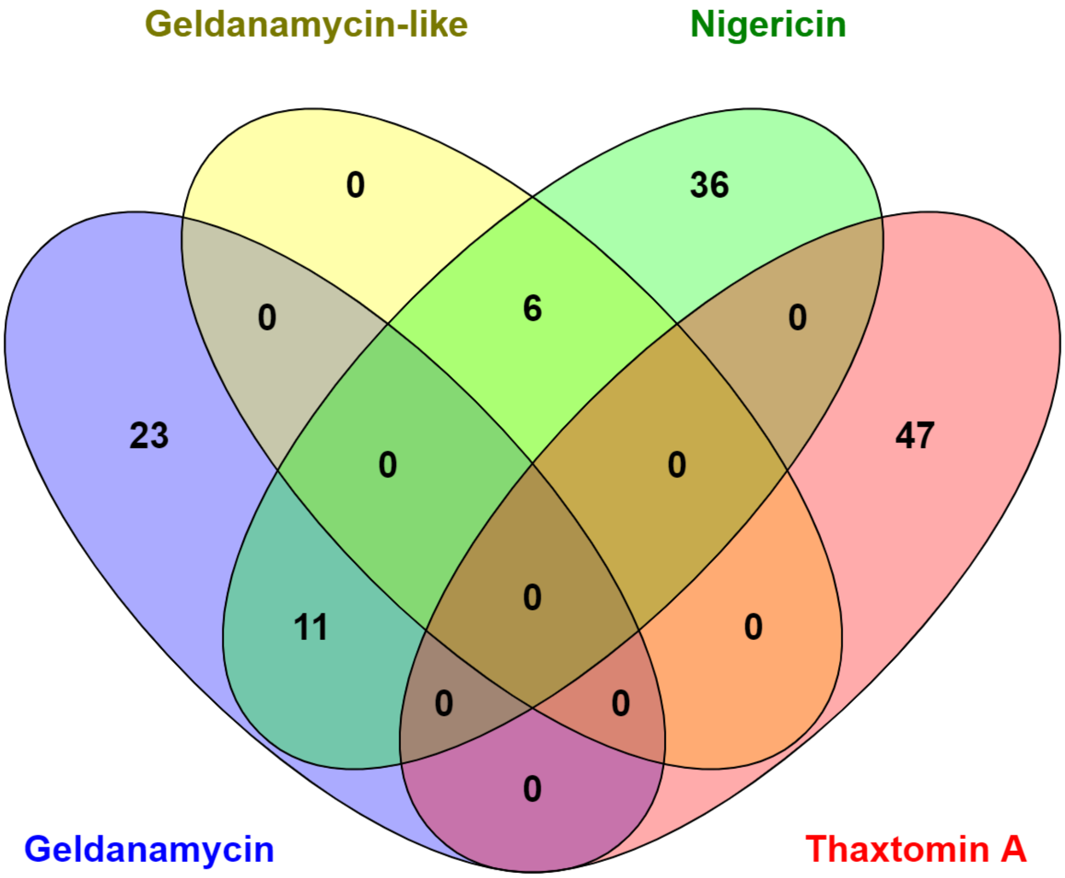


**Fig. S1.** Venn diagram for the distribution of BGCs across the *Streptomyces* genus. The BGC for geldanamycin (GenBank accession AY179507.1.), nigericin (GenBank accession DQ354110.1.) and thaxtomin A (extracted from NC_013929.1) were used to search *Streptomyces* genome sequences on NCBI using the online BLASTn tool with the following parameters: database refseq_genomes, ≥70% identity, ≥70% coverage, and E-values < $10^{-6}$. Matches to the geldanamycin BGC were refined by searching for the presence of genes *gdmF* and *gdmM*. BGCs lacking *gdmF* and *gdmM* but with similarity to the geldanamycin gene cluster were labeled as "Geldanamycin-like". The diagram was prepared using Venny 2.1 (https://bioinfogp.cnb.csic.es/tools/venny/).

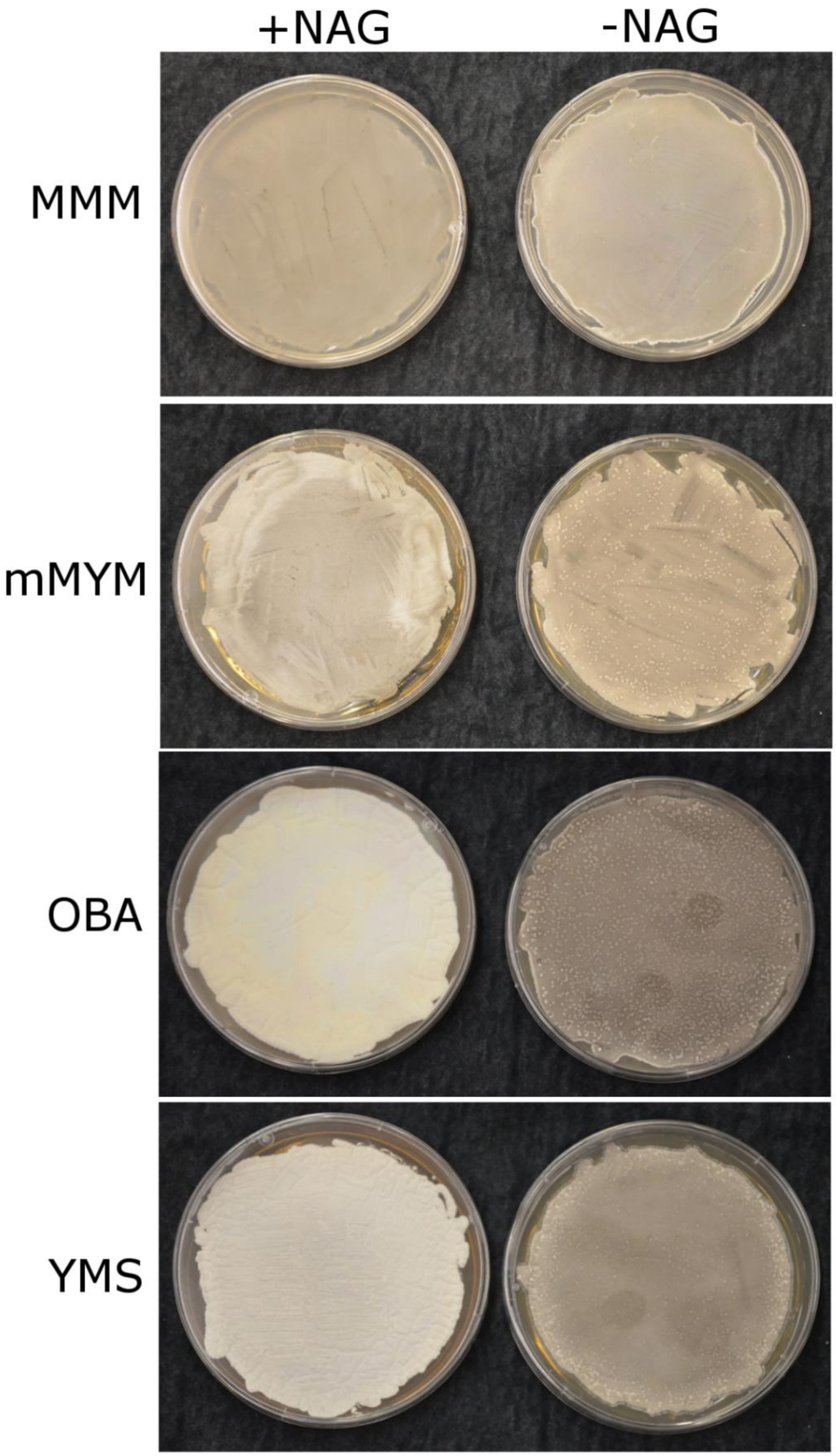


**Fig. S2.** Morphological development of *Streptomyces* sp. 11-1-2 on different culture media containing (+) or lacking (-) 50 mM NAG. Photos were taken 14 days after inoculation and incubation at 28°C.

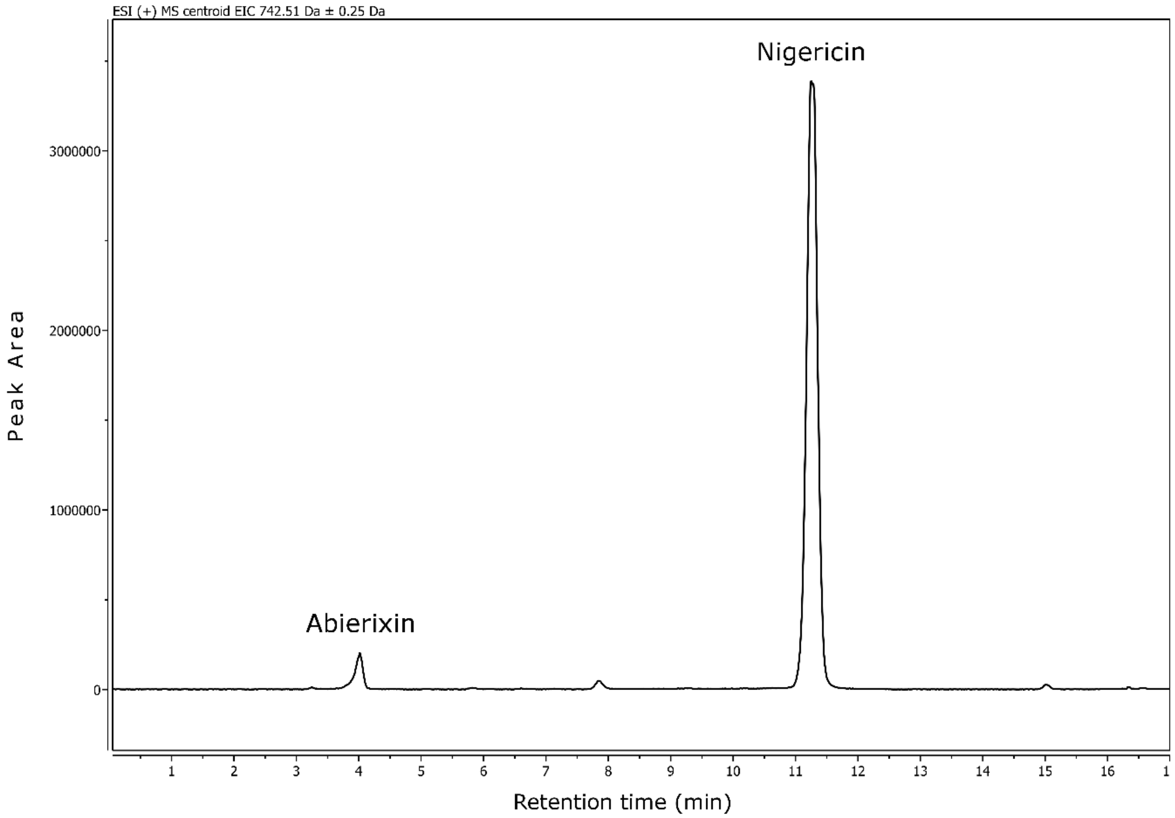


**Fig. S3.** Representative extracted ion chromatogram for nigericin and abierixin. The chromatogram was obtained by analyzing a YMS - NAG extract for the *m/z* 742.51 ($[M+NH_4]^+$) using MestReNova version 14.1.2 (Mestrelab Research S.L.).

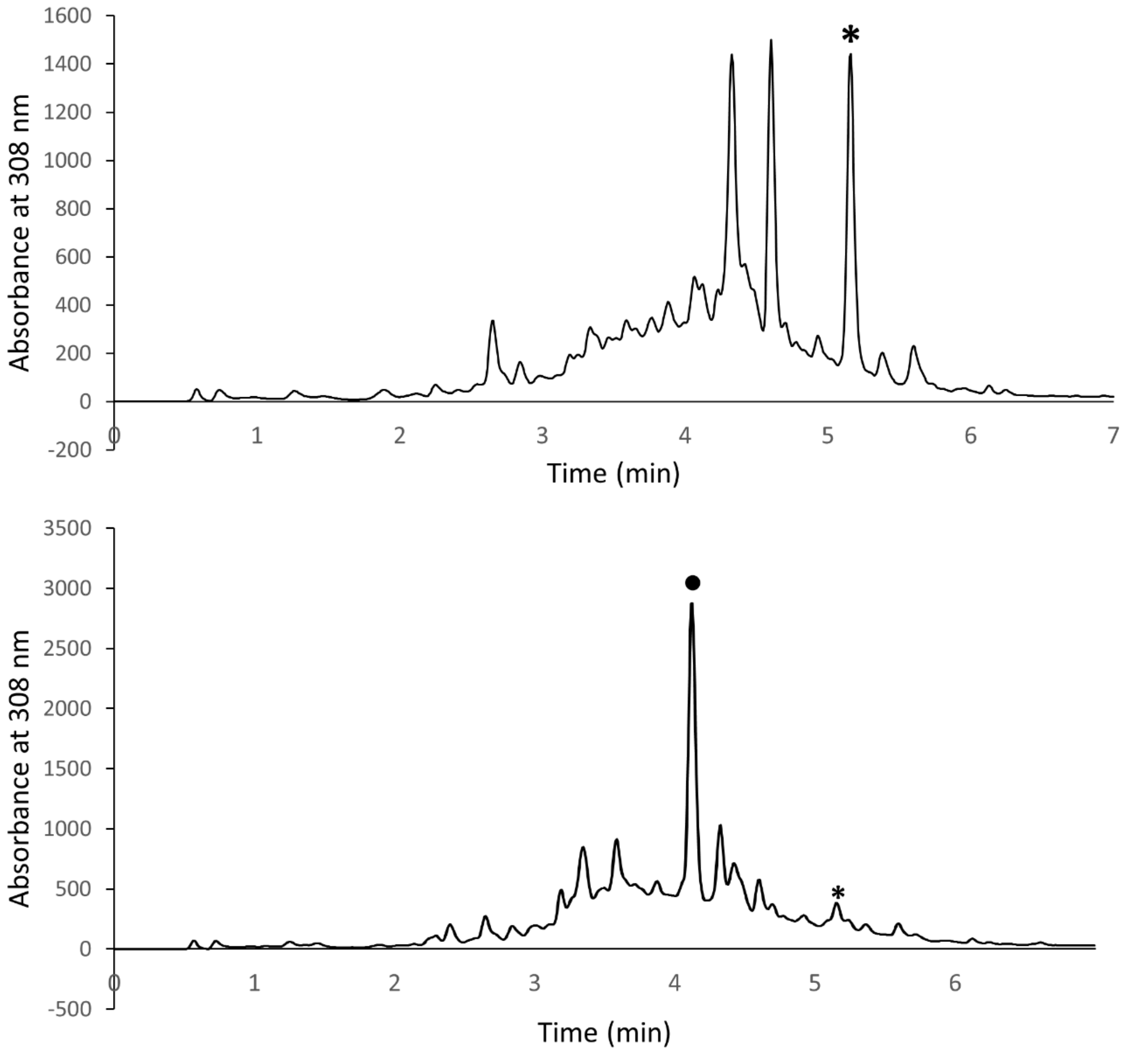


**Fig. S4**. Representative chromatograms for geldanamycin detection using RP-HPLC. The chromatograms correspond to extracts from a plate culture of strain 11-1-2 on YMS - NAG (top) and YMS + 50 mM NAG (bottom). The geldanamycin peak is indicated by *, and the predicted 15-hydroxygeldanamycin peak is indicated by ●.
